# Genome-, Transcriptome- and Proteome-Wide Analyses of the Gliadin Gene Families in *Triticum urartu*

**DOI:** 10.1371/journal.pone.0131559

**Published:** 2015-07-01

**Authors:** Yanlin Zhang, Guangbin Luo, Dongcheng Liu, Dongzhi Wang, Wenlong Yang, Jiazhu Sun, Aimin Zhang, Kehui Zhan

**Affiliations:** 1 College of Agronomy/The Collaborative Innovation Center of Grain Crops in Henan, Henan Agricultural University, Zhengzhou, China; 2 State Key Laboratory of Plant Cell and Chromosome Engineering, Institute of Genetics and Developmental Biology, Chinese Academy of Sciences, Chaoyang District, Beijing, China; 3 University of the Chinese Academy of Sciences, Beijing, China; Murdoch University, AUSTRALIA

## Abstract

Gliadins are the major components of storage proteins in wheat grains, and they play an essential role in the dough extensibility and nutritional quality of flour. Because of the large number of the gliadin family members, the high level of sequence identity, and the lack of abundant genomic data for *Triticum* species, identifying the full complement of gliadin family genes in hexaploid wheat remains challenging. *Triticum urartu* is a wild diploid wheat species and considered the A-genome donor of polyploid wheat species. The accession PI428198 (G1812) was chosen to determine the complete composition of the gliadin gene families in the wheat A-genome using the available draft genome. Using a PCR-based cloning strategy for genomic DNA and mRNA as well as a bioinformatics analysis of genomic sequence data, 28 gliadin genes were characterized. Of these genes, 23 were α-gliadin genes, three were γ-gliadin genes and two were ω-gliadin genes. An RNA sequencing (RNA-Seq) survey of the dynamic expression patterns of gliadin genes revealed that their synthesis in immature grains began prior to 10 days post-anthesis (DPA), peaked at 15 DPA and gradually decreased at 20 DPA. The accumulation of proteins encoded by 16 of the expressed gliadin genes was further verified and quantified using proteomic methods. The phylogenetic analysis demonstrated that the homologs of these α-gliadin genes were present in tetraploid and hexaploid wheat, which was consistent with *T*. *urartu* being the A-genome progenitor species. This study presents a systematic investigation of the gliadin gene families in *T*. *urartu* that spans the genome, transcriptome and proteome, and it provides new information to better understand the molecular structure, expression profiles and evolution of the gliadin genes in *T*. *urartu* and common wheat.

## Introduction

Common wheat (*Triticum aestivum* L.) could be developed into numerous kinds of foods owing to the viscoelastic properties of dough, which makes it one of the “big three” cereal crops in human diets [1, 2]. These viscoelastic properties are conferred by seed storage proteins, including glutenins and gliadins [3]. Gliadins belong to the proline- and glutamine-rich prolamin family and account for approximately 40–50% of the total storage-protein content [4]. Gliadins are alcohol-soluble proteins that can be generally subdivided into α-, β-, γ- and ω- types (α- and β- types are usually combined as α-type due to their highly identical amino acid sequences) based on their mobility in acid polyacrylamide gel electrophoresis (A-PAGE) [5]. However, recently characterized gliadins, including low-molecular-weight (LMW)-gliadins and δ-gliadin, have been difficult to classify due to their specific structures and functionalities [6, 7]. In common wheat, each of the above three common types of gliadin genes (α, γ and ω groups) forms a multigene family whose members are primarily located at the *Gli-1* and *Gli-2* loci on the short arms of the homoeologous group 1 and 6 chromosomes, respectively [8]. The *Gli-1* loci encode all of the ω-gliadins, most of the γ-gliadins and several of the β-gliadins, whereas the genes at the *Gli-2* loci code for all the α-gliadins, most of the β-gliadins, and several of the γ-gliadins [9]. As for the newly identified gliadins, the LMW-gliadin genes are located on group 4 and 7 chromosomes, and the δ-gliadin genes are found in the *Gli-1/Glu-3* region in each of the wheat genomes [6, 7]. Apart from their common signal peptides, each type of gliadin has a unique structure. Mature α-gliadin proteins contain a repetitive domain, two polyglutamine stretches and two unique domains [10]; γ-gliadins are composed of an N-terminal non-repetitive domain (I), a repetitive domain (II), a non-repetitive domain (III), a polyglutamine region (IV) and a C-terminal non-repetitive domain (V) [11], and mature ω-gliadin proteins only contain a N- and C-terminal domains and an intervening repetitive domain [12]. Most α-gliadins contain six conserved cysteine residues, which form intramolecular disulfide bonds, in their two unique domains [10]. In γ-gliadins, eight cysteine residues, all of which also form intramolecular disulfide bonds, have been characterized in the non-repetitive domain II and the C-terminal non-repetitive domain V [11]. However, α- and γ-gliadins containing an odd number of cysteine residues have also been reported, and these gliadins can form intermolecular disulfide bonds and join a gluten polymer [11]. Unlike in the α- and γ-gliadins, cysteines have not been observed in most of the ω-gliadin protein sequences [12]. Because of their cysteines, α- and γ-gliadins are more likely to interact with the gluten matrix than ω-gliadins, and ω-gliadins might interfere with or modify the interaction among the gluten proteins and affect the viscoelastic properties of gluten [13]. Interestingly, ω-gliadins with at least one cysteine were also recently characterized and they might form intermolecular disulfide bonds with other components of the gluten matrix [14]. Despite their vital roles in determining dough quality, gliadins can cause various diseases, such as celiac disease (CD), wheat-dependent exercise-induced anaphylaxis (WDEIA), and baker’s asthma [15–17]. Among the α-gliadins, five epitopes, namely DQ2.5-glia-α1a (PFPQPQLPY), DQ2.5-glia-α1b (PYPQPQLPY), DQ2.5-glia-α2 (PQPQLPYPQ), DQ2.5-glia-α3 (FRPQQPYPQ), DQ8-glia-α1 (also nominated as DQ8.5-glia-α1, QGSFQPSQQ), were found to be relevant to CD [18, 19]. Furthermore, a 19-residue peptide (LGQQQPFPPQQPYPQPQPF) in the N-terminal repetitive domain and a 12-residue peptide (LGQGSFRPSQQN) in unique domain II are also considered to be the cause of CD and adenovirus type 12 infection [20, 21]. With regards to the γ-gliadins, 11 peptides, DQ2.5-glia-γ1 (PQQSFPQQQ), DQ2.5-glia-γ2 (IQPQQPAQL), DQ2.5-glia-γ3 (QQPQQPYPQ), DQ2.5-glia-γ4a (SQPQQQFPQ), DQ2.5-glia-γ4b (PQPQQQFPQ), DQ2.5-glia-γ4c (QQPQQPFPQ), DQ2.5-glia-γ4d (PQPQQPFCQ), DQ2.5-glia-γ5 (QQPFPQQPQ), DQ8-glia-γ1a (QQPQQPFPQ, the same amino acid sequence as DQ2.5-glia-γ4c but deamidation of a glutamine residue at a different position by enzyme transglutaminase 2), DQ8-glia-γ1b (QQPQQPYPQ, the same amino acid sequence as DQ2.5-glia-γ3 but deamidation of a glutamine residue at a different position by enzyme transglutaminase 2) and DQ8.5-glia-γ1 (PQQSFPQQQ, the same amino acid sequence as DQ2.5-glia-γ1 but deamidation of a glutamine residue at a different position by enzyme transglutaminase 2), have been identified as gluten T-epitopes that cause CD [18]. Except the two epitopes [DQ2.5-glia-ω1 (PFPQPQQPF) and DQ2.5-glia-ω2 (PQPQQPFPW)], nine heptapeptides (QQIPQQQ, QQLPQQQ, QQFPQQQ, QQSPQQQ, QQSPEQQ, QQYPQQQ, QSPEQQQ, YQQYPQQ and QQFHQQQ), two tetrapeptides (QQQP and PYPP) and one hexapeptide (QQPPQQ), are the most immunoreactive epitopes of the ω-gliadins [18, 22–24].

The gliadin genes have generally been isolated from genomic DNA, expressed sequence tag (EST) and bacterial artificial chromosome (BAC) libraries [25–27]. However, the identification of the full complement of gliadin gene family members in common wheat and its relatives remains challenging because of the large sizes of these gene families, the similar genetic structure within a family and the lack of genomic data [28]. Hexaploid wheat and ancestral species are thought to contain between 25 and 150 α-gliadin genes [29–32] and between 15 and 50 γ-gliadin genes [33]; and only a limited number of ω-gliadin genes have been reported [12, 14]. Moreover, most of the known α-gliadin genes (72–95%) are pseudogenes that contain varying numbers of in-frame stop codons [10, 34]. The ω-gliadin gene family also contains a large proportion of pseudogenes; however, only a small proportion of γ-gliadin genes (approximately 14%) in hexaploid wheat are pseudogenes [35].

The tremendous genetic variability of wild wheat ancestors and related species makes them ideal genetic reservoirs for common wheat breeding [36]. In recent decades, these genetic resources have been used to characterize numerous important agronomic genes (i.e., *Sr47*, *Lr41-43*, *Gpc-B1*) [37–39]. *Triticum urartu* is a wild diploid wheat species that is distributed in the Fertile Crescent, and it has long been considered the A-genome donor of polyploid wheat species [40]. Recently, a set of genes that includes a powdery mildew resistance gene (*PmU*) and grain-length-controlling gene (*TuGASR7*) was characterized in *T*. *urartu* [41, 42]. Numerous glutenin gene variants have also been detected in *T*. *urartu* using electrophoretic procedures and nucleotide sequence analysis [43, 44]. Although A-PAGE has been used to detect abundant and variable gliadin proteins in *T*. *urartu*, only a small percentage of gliadin genes have been isolated from this species to date [31, 45, 46] when considering the huge number of family members [29–33]. The *T*. *urartu* genome (PI428198) was recently sequenced, and the genomic data could facilitate the comprehensive identification of gliadin gene family members in this species [41].

In past decades, several hundred gliadin gene sequences have been cloned from hexaploid wheat and deposited in GenBank. However, these sequences were cloned from a variety of germplasms, and the data are far from fully representing the entire composition of gliadin gene families in hexaploid wheat, even though a significant number of gliadin genes were cloned from a few varieties, eg. Jimai 20, Jinan 177, and Yumai 34 (http://www.ncbi.nlm.nih.gov). Moreover, due to the extremely high level of sequence similarity among the genes and the similar molecular weights (MWs) and isoelectric points (pIs) of their protein products, the dynamic expression patterns of gliadin genes during grain development and their protein product accumulation in seeds have seldom been investigated [47–49]. In the present study, the genome sequence of *T*. *urartu* accession PI428198 was used in concert with genomic DNA and cDNA to characterize the gliadin genes in this species. Using RNA-sequencing (RNA-Seq) technology, the digital expression patterns of these gliadin genes were investigated during grain development. The protein products of these genes were separated by two-dimensional electrophoresis (2-DE) and further assayed using matrix-assisted laser desorption/ionization time-of-flight tandem mass spectrometry (MALDI-TOF/TOF-MS) or liquid chromatography-tandem mass spectrometry (LC-MS/MS). Furthermore, a phylogenetic and evolutionary analysis of the α-gliadin genes was conducted. Our work offers a systematic investigation of the gliadin gene families in *T*. *urartu* at the genomic, transcriptomic and proteomic levels, and it provides new information to further understand the molecular structure, expression profiles and evolution of the gliadin genes in *T*. *urartu* and common wheat.

## Materials and Methods

### Plant materials

The *T*. *urartu* accession (PI428198) was grown at the experimental station of the Institute of Genetics and Developmental Biology of the Chinese Academy of Sciences (IGDB, CAS), Beijing, China. Young leaves were collected from 30-day-old seedlings for genomic DNA extraction. For RNA-Seq and quantitative reverse transcription PCR (qRT-PCR) analysis, developing seeds from the central part of four spikes were harvested at 5, 10, 15, 20 and 25 days post-anthesis (DPA). As a control for the transcriptomic analysis, flag leaves were collected at 10 DPA. The endosperm and leaf samples were immediately frozen in liquid nitrogen and stored at -80°C.

### DNA isolation, RNA extraction and cDNA synthesis

Genomic DNA was isolated from the young leaves of 30-day-old seedlings using the cetyltrimethylammonium bromide (CTAB) method [50]. Total RNA from individual grain samples at different developmental stages as well as from the flag leaves was extracted using a quick method designed specifically for grains with high starch content [51]. The extracted total RNA was dissolved in diethylpyrocarbonate (DEPC)-treated water, and the contaminating genomic DNA was removed using RNase-Free DNase (Promega, Madison, WI, USA). The RNA quality was assessed using a bioanalyzer (Agilent, Palo Alto, CA, USA), and only those samples with RNA integrity number (RIN) scores greater than 8.0 were used to construct libraries for RNA-Seq. Three biological replicates were performed for each developmental stage of the grain. For gene cloning and qRT-PCR, cDNA was synthesized from approximately 3 μg total RNA using Quant Reverse Transcriptase (Tiangen Biotech, Beijing, China), and the cDNA was diluted to 100 μl for further PCR analysis.

### PCR amplification and sequence analysis

To characterize the gliadin genes in *T*. *urartu* via PCR-based methods, all of the gliadin gene sequences in GenBank were downloaded (before 2014-7-20) and aligned using Lasergene software (DNASTAR; http://www.dnastar.com/). This analysis resulted in the design of seven conserved PCR primers flanking the coding sequences of the gliadin genes, with three primers each for the α- and γ-gliadin genes and one primer for ω-gliadin (S1 Table). The PCR reactions were performed at a volume of 20 μl, which contained 100 ng genomic DNA or 2 μl diluted cDNA, 4 pmol forward and reverse primers, and 4 nmol (each) deoxynucleotide (dNTP), 1× GC buffer I (Mg^2+^, plus) and 1.0 U LA-Taq DNA polymerase (Takara Bio, Otsu, Japan). PCR amplification was conducted using a PTC-220 DNA Engine Dyad Peltier Thermal Cycler (Bio-Rad, Hercules, CA, USA) with the following conditions: 95°C for 5 min; followed by 35 cycles of 94°C for 30 s, 55 or 61°C for 30 s, 72°C for 90 s; and a final extension step at 72°C for 10 min.

To search gliadin gene sequences in the PI428198 genome sequence database (http://gigadb.org/dataset/100050) [41], representative sequences of gliadin, avenin-like, hordein and secaline genes available in GenBank (S2 Table) were used as query sequences in the basic local alignment search (ftp://ftp.ncbi.nlm.nih.gov/blast/executables/LATEST) with the manufacturer’s E-value and identity threshold. Additionally, the subjective sequences from the database were further confirmed as gliadin genes or not based on the features of their predicted amino acid sequences. The gliadin gene sequences were assembled and aligned using Lasergene software, and the amino acid residues encoded by these sequences were visualized using GeneDoc (http://www.nrbsc.org/gfx/genedoc/). The previously characterized epitopes and immunoreactive oligopeptides were assigned back to the predicted amino acid sequences of gliadin genes characterized in this work with 100% match [18–24].

To analyze the phylogenetic relationships between the α-gliadin genes in *T*. *urartu* characterized in this work and their orthologs in related species, all the α-gliadin genes available in GenBank from *Triticum monococcum*, *Aegilops speltoides*, *Aegilops tauchii*, *Triticum dicoccoides*, *Triticum durum* and *T*. *aestivum* were downloaded. The α-gliadin genes from other *T*. *urartu* accessions available in GenBank were also downloaded to expand the gene diversity of *T*. *urartu*. These nucleotide sequences were aligned using ClustalW2 (http://www.ebi.ac.uk/Tools/msa/clustalw2), and one sequence was chosen as the representative in each clade from the phylogenetic tree displayed on the ClustalW2 website. Finally, 66 α-gliadin genes were selected, including ten in *T*. *monococcum*, ten in *Ae*. *speltoides*, 11 in *Ae*. *tauschii*, eight in *T*. *dicoccoides*, two in *T*. *durum*, 13 in *T*. *aestivum*, and 12 in other *T*. *urartu* accessions, and their deduced amino acid sequences with polyglutamine domains I and II deleted were subjected to the phylogenetic tree construction using the neighbor-joining method with 1000 bootstrap replications and the MEGA5 software [52].

### RNA-Seq and qRT-PCR

RNA-Seq libraries were prepared using a NEXTflex Directional RNA-Seq Kit (dUTP-based) (Bioo Scientific, Austin, TX, USA) according to the manufacturer’s protocol. Briefly, the mRNA was concentrated using oligo(dT) magnetic affinity adsorption, and then sheared into fragments as templates to synthesize first- and second-strand cDNA. The double-stranded cDNA was purified using a QiaQuick PCR extraction kit (Qiagen, Hilden, Germany), resolved for end repair and poly(A) addition, and then ligated to different sequencing adapters. A library with an insert length of approximately 350 bp was sequenced, and approximately 100-bp paired-end reads were generated using the Illumina HiSeq 2000 (San Diego, CA, USA) at the Genomic Analysis Platform at the IGDB, CAS (Beijing, China). After filtering out the adaptor sequences and the low-quantity reads (more than 50% of bases with Q-values ≤10) using TopHat v. 2.0.10, the remaining high-quality reads were aligned back to the gliadin gene sequences characterized using the PCR cloning method described above. The expression levels of the gliadin genes were quantified using the reads per kilobase per million mapped reads (RPKM) value, which was calculated using the uniquely aligned reads of each gene with 100% identity.

The RNA-Seq data were validated by qRT-PCR analysis of a set of gliadin genes (S3 Table). qRT-PCR was performed on a Roche LightCycler 480 system with a SYBR Green I Master Kit (Roche, Basel, Switzerland) [53]. Each 10-μl reaction contained 5 μl SYBR green I mix, 2 μl cDNA template and 3 μl forward and reverse primers (1 μM) (S3 Table). For each gliadin gene, three biological replicates and three technical replicates were performed for each sample. *Ta4050* (Ubiquinol-cytochrome C reductase iron-sulfur subunit, http://compbio.dfci.harvard.edu/tgi/cgi-bin/tgi) was used as the reference gene with the primers Ta4050F (5’-CCTGCCCCGTACAACCTTGAG-3’) and Ta4050R (5’-CACCGTTGCGATAGTCCTGAAAC-3’) [54]. The relative gene expression levels were estimated by comparing the threshold cycles of the target genes with those of *Ta4050*. For each target gene, a pair of specific primers was designed whose qRT-PCR product had a unique melting temperature and was of the expected length as shown by gel electrophoresis. The primer specificity was also confirmed by sequencing the qRT-PCR products.

### Two-dimensional electrophoresis and MALDI-TOF/TOF-MS

For the 2-DE, the gliadins were extracted in a stepwise manner from 0.1 g flour samples prepared from *T*. *urartu* PI428198 [55]. The albumins and globulins present in the supernatant after centrifugation at 15,000 g and 20°C for 5 min were removed and discarded three times using 1.0 ml 0.4 M NaCl and 67 mM KH_2_PO_4_ (pH 7.6), and the final pellets were dissolved in 0.6 ml 70% v/v ethanol through homogenization at 65°C for 30 min. Subsequently, the homogenized sample was centrifuged at 15,000 g and 20°C for 5 min, and the supernatants were lyophilized for 40 min with LGJ-12 Freeze Dryer from Beijing Songyuanhuaxing Science and Technology Development Co., Ltd., China. The resulting gliadin pellet was dissolved in isoelectric focusing (IEF) sample extraction solution [containing 50 mM dithiothreitol (DTT), 0.5% v/v immobilized pH gradient (IPG) buffer (pI 3–10), 4% w/v 3-[(3-cholamidopropyl)-dimethylammonio]-1-propanesulfonate (CHAPS), and 8.0 M urea] and the gliadin concentration was quantified with the 2D Quant Kit according to the manufacturer’s instructions (GE Healthcare, Buckinghamshire, UK).

IEF and 2-DE were performed mainly following previously described methods [56]. A 24-cm immobilized strip (GE Healthcare, Buckinghamshire, UK), with a linear pH gradient from 3 to 10, was subjected to strip rehydration with a volume equivalent to 350 μg gliadin extract, and the 2-DE images were analyzed using ImageMaster 2D Platinum software (Version 6.0, GE Healthcare, Buckinghamshire, UK). The pIs, MWs, and spot volumes of the separated proteins were calculated. The spots on the 2-DE gel were recovered and digested with chymotrypsin (Sigma-Aldrich, MO, USA), and the peptides were prepared. Most of the peptides (except for spots 15 and 16) were then subjected to a MALDI-TOF/TOF-MS analysis (AB SCIEX 5800), and the data were analyzed using the MASCOT 2.0 search engine (Matrix Science, London, U.K.) and searched against the National Center for Biotechnology Information (NCBI) database (accessed prior to 2014–7) consisting of *Triticeae* protein sequences and against amino acid sequences predicted from the genomic data of *T*. *urartu* (http://gigadb.org/dataset/100050). The peptide mass tolerance and MALDI-TOF/TO F-MS ion tolerance were set to 0.2 Da and 0.5 Da, respectively, and protein scores greater than 58 were considered significant (p<0.05). The peptides extracted from spots 15 and 16 were analyzed via LC-MS/MS and Bioworks 3.1 software using the same protein databases as in the MALDI-TOF/TOF-MS analysis [56]. The peptides identified as gliadins in the above database search were subsequently matched to the predicted protein sequences of the gliadin genes characterized in this work.

## Results

### Molecular characterization of the gliadin gene families in *T*. *urartu*

To comprehensively characterize the gliadin gene families in *T*. *urartu*, a PCR-based cloning method was employed to isolate gliadin sequences from the genomic DNA and cDNA of developing grains at 15 and 20 DPA using conserved primers designed for the α, γ and ω fractions. The PI428198 draft genome sequences were analyzed, and nine gliadin gene family members were incorporated into the design of the conserved primers. Ultimately, 23 α-, three γ-, and two ω-gliadin genes were identified in the genomic DNA of PI428198, designated as *Gli-α-1* ~ *Gli-α-23*, *Gli-γ-1* ~ *Gli-γ-3*, and *Gli-ω-1* ~ *Gli-ω-2* (Table 1). Of the 28 genes, 12 of them could be detected in the cDNA of immature grains, including eight α-, three γ-, and one ω-gliadin genes, whose corresponding genomic DNA sequences contained an uninterrupted full open reading frame (full ORF) in further analysis (Table 1). Based on sequence identity, the nine gliadin genes found in the draft genome were among those characterized using the PCR-based cloning strategy, thus demonstrating the efficiency of this gene cloning method and suggesting that the genomic data are insufficient. All the cloned gliadin gene sequences were deposited in GenBank with accession numbers from KP280176 to KP280203 (Table 1).

**Table 1:** Gliadin genes of *T*. *urartu* accession PI428198.

**Gene** ^a^	**GenBank ID**	**cDNA** ^b^	**Genome ID** ^c^	**Fragment length** ^d^	**Predicted amino acid length** ^e^	**Number of cysteines**
***Gli-α-1***	KP280186	N	NA	852	*pseudo*	6
***Gli-α-2***	KP280187	Y	NA	843	281	6
***Gli-α-3***	KP280197	Y	NA	834	278	6
***Gli-α-4***	KP280198	N	NA	906	302	6
***Gli-α-5***	KP280199	Y	TRIUR3_32056	858	286	6
***Gli-α-6***	KP280200	N	NA	876	292	6
***Gli-α-7***	KP280201	Y	NA	885	295	6
***Gli-α-8***	KP280202	Y	NA	855	285	6
***Gli-α-9***	KP280203	N	NA	864	288	6
***Gli-α-10***	KP280176	N	TRIUR3_24642	909	303	6
***Gli-α-11***	KP280177	Y	NA	903	301	6
***Gli-α-12***	KP280178	Y	NA	882	294	6
***Gli-α-13***	KP280179	Y	TRIUR3_24643	864	287	6
***Gli-α-14***	KP280180	N	TRIUR3_35338	833	*pseudo*	6
***Gli-α-15***	KP280181	N	NA	786	*pseudo*	5
***Gli-α-16***	KP280182	N	NA	786	*pseudo*	5
***Gli-α-17***	KP280183	N	NA	850	*pseudo*	7
***Gli-α-18***	KP280184	N	NA	847	*pseudo*	7
***Gli-α-19***	KP280185	N	NA	841	*pseudo*	7
***Gli-α-20***	KP280188	N	TRIUR3_28870	848	*pseudo*	6
***Gli-α-21***	KP280189	N	TRIUR3_34337	858	*pseudo*	6
***Gli-α-22***	KP280190	N	TRIUR3_28127	885	*pseudo*	6
***Gli-α-23***	KP280191	N	TRIUR3_29675	905	*pseudo*	6
***Gli-γ-1***	KP280192	Y	NA	858	286	8
***Gli-γ-2***	KP280193	Y	NA	837	279	8
***Gli-γ-3***	KP280194	Y	TRIUR3_27774	1026	342	8
***Gli-ω-1***	KP280195	Y	NA	975	325	0
***Gli-ω-2***	KP280196	N	NA	1248	*pseudo*	0

^a^α, γ, and ω represents alpha-, gamma- and omega-gliadin genes, respectively

^b^Y indicates that gene sequences were cloned from the cDNA of both immature grain stages (15 and 20 DPAs), and N not cloned

^c^Genome ID in the *T*. *urartu* draft genome (Ling et al. 2013); in this column, NA indicates that homologs of these gliadin genes could not be detected in the draft genome

^d^Length of the putative full open reading frame in base pairs (bps);

^e^Length of the predicted amino acid sequences of the gliadin genes with full open reading frames; *pseudo* represents pseudogenes.

### α-gliadin genes

**Fig 1:**
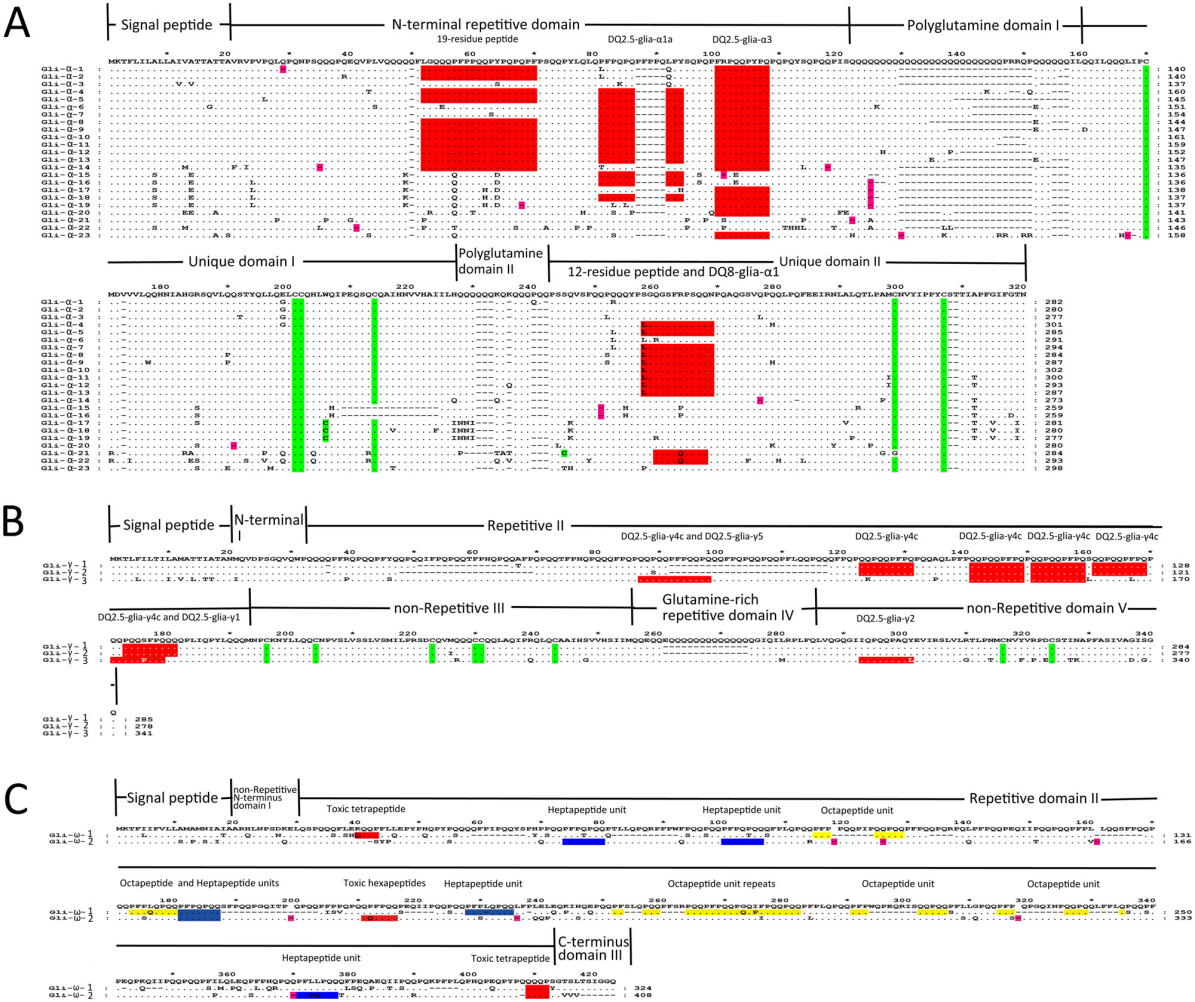
Multiple alignment of the predicted amino acid sequences of the gliadin genes from *T*. *urartu*. (A) Protein sequence alignment of the 23 α-gliadin genes. (B) Protein sequence alignment of the three γ-gliadin genes. (C) Protein sequence alignment of the two ω-gliadin genes. The stop codons are indicated by dashes with a pink background, and deletions are dashes without any background. The cysteine residues and toxic epitopes in each type of gliadin were highlighted by green and red backgrounds, respectively. The heptapeptide units in the ω-gliadins have a blue background, and the octapeptide units have a yellow background.

All 23 α-gliadins (Gli-α-1 to Gli-α-23) displayed typical structural features, including a short signal peptide, N-terminal repetitive domain, two polyglutamine domains (QI and QII) and two unique domains (UI and UII) (Fig 1A). Although the α-gliadin genes belong to a large gene family and share high similarities, they are still highly diverse because of their abundant insertion/deletion (indel) variations and various single nucleotide polymorphisms (SNPs). The 23 α-gliadin genes varied greatly in length, with the shortest, *Gli-α-15* and *Gli-α-16*, at 786 bp long and the longest, *Gli-α-10*, at 909 bp long; in addition, only four pairs of gliadin genes had identical sequence lengths (Fig 1A and Table 1). Of the typical structures in the α-gliadins, the two polyglutamine domains were the most diverse, and length variations were largely caused by indels (Fig 1A). The nucleotides encoding the QI residues varied in length from 27 bp to 99 bp, and these nucleotides accounted for most of the differences in length of the α-gliadin genes (Fig 1A and S1 Fig). Other indels scattered in the N-terminal repetitive domain and QII, UI and UII domains also contributed to the sequence-length polymorphisms (Fig 1A and S1 Fig). However, the regions flanking the QI were relatively conserved, especially the signal peptide and UI regions, and their corresponding nucleotide sequences were also more similar across all α-gliadin genes (Fig 1A and S1 Fig). This phenomenon facilitated the development of conserved PCR primers targeted to the signal peptide and UI regions, amplification of all α-gliadin gene fragments and characterization of the α-gliadin gene lengths using high-resolution capillary electrophoresis, which was attempted by the molecular marker system used for low-molecular-weight glutenin subunit genes in common wheat [57].

Twelve of the 23 α-gliadin genes (*Gli-α-2* to *Gli-α-13*) contained an uninterrupted full open reading frame (full ORF), and the remaining sequences contained between one and three internal stop codons or a frameshift mutation, which were referred to as pseudogenes (Fig 1A). To determine how these pseudogenes evolved, their internal stop codon positions and relationships with intact ORFs in the same loci were determined. The pseudogenes were structurally similar to the full-ORF genes, and its internal stop codons were mainly located at the positions of glutamine codons in the full-ORF genes, which is a similar result to that of the pseudo-gliadin genes in *T*. *monococcum* (Fig 1A) [34]. Stop codons in the QI domain were prevalent, and more than 50% of the pseudogenes contained a stop codon in this domain, whereas the other internal stop codons were scattered throughout the sequences encoding all of the other domains except the signal peptide (Fig 1A). A comparison of the full-ORF genes showed that the stop codon was created in all cases by a C-to-T substitution that changed a CAG or CAA codon for glutamine or (in one case) a CGA codon for arginine into a TAG, TAA or TGA stop codon (Fig 1A and S1 Fig). Only the *Gli-α-23* pseudogene contained a frameshift mutation, wherein the A at bp 471 was deleted, resulting in the generation of a stop codon (Fig 1A and S1 Fig). Cysteine skeleton, which form intra- and inter-molecular disulfide bonds, is widely accepted as the key feature of gliadins. Most of the α-gliadins contained six highly conserved cysteines, with four in the UI domain and two in the UII domain. The exceptions were Gli-α-15 and Gli-α-16, which each contained five cysteines, and Gli-α-17, Gli-α-18 and Gli-α-19, with seven cysteins each (Fig 1A). In the UI, a 16-AA sequence, containing the fourth cysteine (in the full-ORF genes), was lacking in Gli-α-15 and Gli-α-16, while a G-to-T change that converted a TGG codon for tryptophan into a TGT codon for cysteine resulted in an additional cysteine in Gli-α-17, Gli-α-18 and Gli-α-19 (Fig 1A and S1 Fig). In Gli-α-21, point mutations changed the position of the fifth cysteine from amino acid 264 to 210, with glycine replacing cysteine at position 264 and cysteine replacing serine at position 210 (Fig 1A). Unfortunately, all of the α-gliadin genes with irregular cysteines were pseudogenes.

CD is caused by T-cell responses to wheat gluten-derived peptides. The presence of such peptides in α-gliadins has been well studied; the 12-residue peptide and DQ8-glia-α1 epitope are found in the UII domain, and the 19-residue peptide and DQ2.5-glia-α1a and DQ2.5-glia-α3 epitopes are present in the N-terminal repetitive region [20, 21, 58, 59]. The number and position of these CD-toxic epitopes, including DQ8-glia-α1, DQ2.5-glia-α1a, DQ2.5-glia-α3, 12-residue peptide and the 19-residue peptide, varied among the 23 cloned α-gliadin sequences (Fig 1A). A majority of the full-ORF genes contained two or four epitopes, whereas Gli-α-3 had only one epitope (DQ2.5-glia-α3), and many pseudogenes also had only one epitope (Fig 1A and S4 Table). The most widespread epitope was DQ2.5-glia-α3, which was present in all 12 of the full-ORF genes and seven of the 11 pseudogenes. The DQ2.5-glia-α1a epitope could be observed in most of the full-ORF genes, and the DQ2.5-glia-α2 epitope was absent from all of the cloned α-gliadin genes, which is consistent with previous data showing that gliadin genes from A-genome species lack the DQ2.5-glia-α2 epitope (Fig 1A and S4 Table) [34]. Interestingly, Gli-α-21 and Gli-α-22 contained the DQ8-glia-α1 epitope, which is inconsistent with the hypothesis that DQ8-glia-α1 is only present in the wheat B-genome (Fig 1A and S4 Table) [34]. The 12-residue peptide was only detected in the UII domains of full-ORF genes, and the majority of the 19-residue peptides were also identified almost exclusively in the N-terminal repetitive domains of the full-ORF genes; however, the *Gli-α-1* and *Gli-α-14* pseudogenes also possessed the 19-residue peptide (Fig 1A and S4 Table). A detailed analysis of these gliadin genes revealed a SNP which caused an amino acid substitution in a specific epitope (Fig 1A), was present in most of them, For example, in 21 of the gliadin genes, the eighth amino acid of the DQ2.5-glia-α2 epitope was mutated and contained a serine (S) instead of a proline (P). In the UII domains of most of the α-gliadins, the DQ8-glia-α1 epitope was disrupted at the fifth amino acid by the substitution of an arginine (R) for a glutamine (Q).

### γ-gliadin genes

Three γ-gliadin genes (*Gli-γ-1* to *Gli-γ-3*) with intact ORFs were characterized in PI428198, and their predicted protein sequences reflected the typical structure of γ-gliadins, including a 20-residue signal peptide and the I-V domains (Fig 1B) [27]. The presence of eight cysteine residues, with six in domain III and two in domain V, leads to the formation of intramolecular disulfide bonds that are responsible for the folded structure of gliadins (Fig 1B), which further determines the nature of their non-covalent bonds [60]. The numerous PFPQ_1-2_(PQQ)_1-2_ repeat units in repetitive domain II [27] and indels in the glutamine-rich domains II and IV were the primary causes of variation in the length of the γ-gliadins. The CD toxic epitopes were also investigated in repetitive domain II and non-repetitive domain IV of the three γ-gliadins [61]. The DQ2.5-glia-γ4c epitope, which had not been detected in the γ-gliadins from A genome in common wheat, was widely distributed in the genome of *T*. *urartu*, for its four repeats were present in the repetitive domains II of all the three γ-gliadins [62]. And one DQ2.5-glia-γ5 epitope and one DQ2.5-glia-γ2 epitope were found in the repetitive domain II and non-repetitive domain IV of Gli-γ-3, respectively (Fig 1B). SNPs were responsible for the absence of DQ8-glia-γ1b (the seventh amino acid, Y, was changed to F), DQ2.5-glia-γ3 (the seventh amino acid, Y, was changed to F), DQ2.5-glia-γ4a (the first amino acid, S, was changed to H/Q/P) and DQ2.5-glia-γ4b (the first amino acid, P, was changed to H/Q/P) in the three γ-gliadins. And DQ2.5-glia-γ4d, which was dominant in the A genome of common wheat, was eliminated from the three γ-gliadins in *T*. *urartu*, for its eighth amino acid, C, mutated to S in Gli-γ-3, and its first P and eighth S changed to R and Y in both Gli-γ1 and Gli-γ-2, respectively (Fig 1B) [62].

### ω-gliadin genes

Although their predicted protein sequences reflected the representative structure of ω-gliadins (signal peptide, non-repetitive N-terminal sequence, repetitive region and C-terminus), *Gli-ω-1* and *Gli-ω-2* shared relatively low sequence identity (76.50%) because of the presence of SNPs throughout their sequences and numerous indels in both their repetitive regions and C-termini (Fig 1C). Neither of these two genes contained cysteine residues. Gli-ω-2 and Gli-ω-1 contained one and two toxic tetrapeptides (QQQP), respectively, in their repetitive regions. And two of the immunoactive hexapeptides (QQFPQQ) were present in the repetitive region of Gli-ω-2 (Fig 1C). Based on the first three amino acids of the mature proteins, Gli-ω-1 appeared to be of the ARQ/E type, whereas the ARH sequence at the start of the mature Gli-ω-2 protein did not match any previously characterized sequences [63]. However, the overall ratio of the Q, P, and F residues in both of these ω-gliadins was 4:3:1, which is the typical ratio for the ARQ/E type. Gli-ω-1 and Gli-ω-2 contained two and four repeated heptapeptide units (PFPQPQQ), respectively, and Gli-ω-1 contained seven repeat octapeptide units (PFPQQPQQ). Interestingly, Gli-ω-1 contained a methionine residue in its repetitive region, which is seldom found in ω-gliadin genes (Fig 1C) [64].

### Transcriptional profiles of the gliadin genes during grain development

Three RNA samples from independent biological replicates of the five grain filling stages (5, 10, 15, 20 and 25 DPA) were used for RNA-Seq analysis, and the flag leaves at 10 DPA were used as controls. After filtering out the adaptor sequences and low-quantity reads, a total of 392.02 million reads were obtained, with an average of 21.78 million reads per sample (library) (S5 Table). The raw reads from each library were independently mapped to the gliadin genes characterized in this work, and the uniquely aligned read counts of each gliadin gene were adjusted for RPKM.

**Fig 2:**
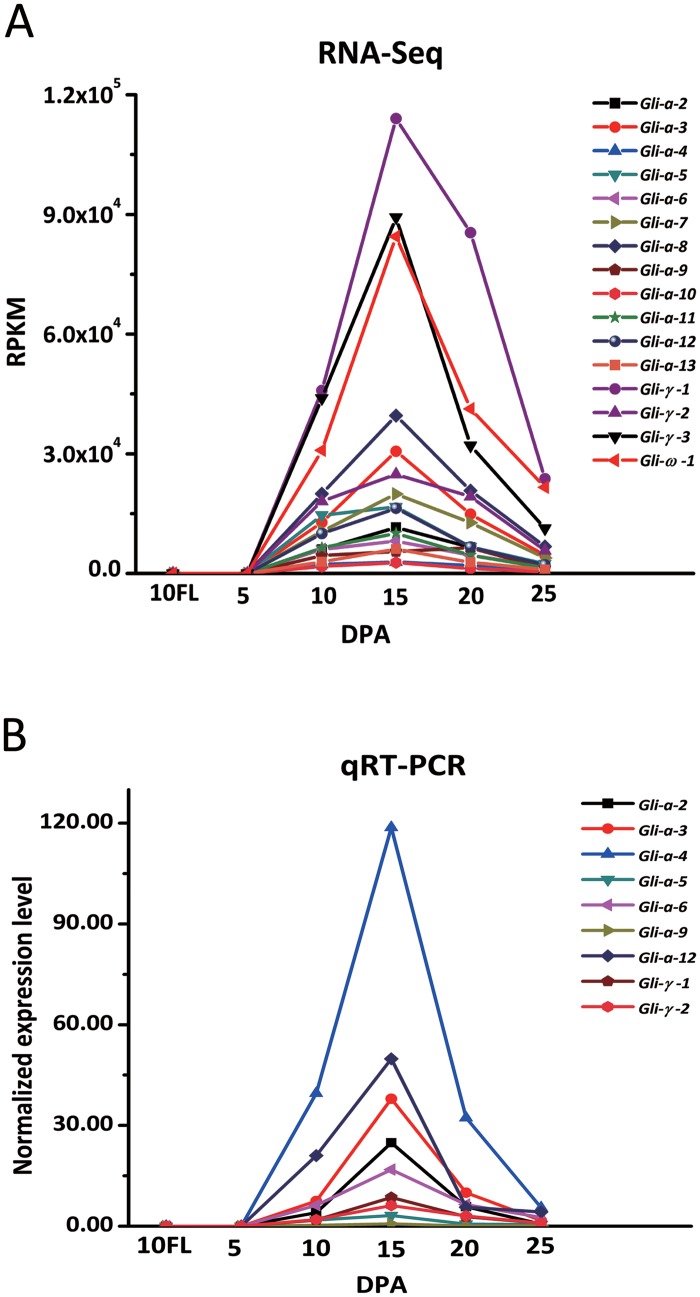
Expression patterns of the *T*. *urartu* gliadin genes as shown by RNA-Seq and qRT-PCR. (A) The expression levels (RPKM values) calculated from the RNA-Seq data. (B) The normalized expression levels, as determined using qRT-PCR.

Of the 28 gliadin genes, 12 α-gliadins (*Gli-α-2* through *Gli-α-13*), three γ-gliadins (*Gli-γ-1* through *Gli-γ-3*), and one ω-gliadin (*Gli-ω-1*) had RPKM values greater than 1000 in samples from various stages (Fig 2A and S6 Table), which indicated that they were expressed; these data were confirmed by qRT-PCR and observations that these genes contained uninterrupted full ORFs. The RPKM values of most of the expressed genes displayed a similar trend wherein they were up-regulated after flowering and down-regulated approaching maturity. In the samples of grains at 5 DPA and flag leaves at 10 DPA, these genes were barely detectable, but their expression was rapidly up-regulated between 5 and 10 DPAs, with an average RPKM value of 14780.45. The expression peaked at 15 DPA and then gradually decreased at 20 and 25 DPAs. The average RPKM value of all the expressed genes at 15 DPA was six-fold the value at 25 DPA and approximately two-fold the value at 10 and 20 DPAs. This expression pattern was observed for most of the gliadin genes except *Gli-α-4*, *Gli-α-5*, *Gli-α-6*, *Gli-α-9*, and *Gli-γ-2*. The expression levels of these five genes at 10 and 20 DPAs were similar to those at 15 DPA (in most cases, more than 70% of the expression level at 15 DPA), which indicated that these gliadin genes were expressed more stably during grain filling (Fig 2A and S6 Table). Generally, the γ- and ω-gliadin genes had higher RPKM values than those of most of the α-gliadin genes. The γ-gliadin gene *Gli-γ-1* had the highest RPKM value at every time point, with a maximum value of 114039.01 at 15 DPA, which was approximately three-fold that of the most highly expressed α-gliadin gene, *Gli-α-8* (39559.93). The only expressed ω-gliadin gene, *Gli-ω-1*, had the second highest RPKM value at most time points, with values of 84444.73, 41230.85 and 21629.78 at 15, 20 and 25 DPAs, respectively. Compared with the expression levels of the γ-gliadin genes, the α-gliadin genes showed significant variation; at 15 DPA, two genes (*Gli-α-3* and *Gli-α-8*) were highly expressed (RPKM values of more than 30000); five genes (*Gli-α-2*, *Gli-α-5*, *Gli-α-7*, *Gli-α-11* and *Gli-α-12*) were moderately expressed (RPKM values between 10000 and 20000); and five genes (*Gli-α-4*, *Gli-α-6*, *Gli-α-7*, *Gli-α-10* and *Gli-α-13*) had much lower RPKM values (less than 10000). At 20 DPA, the highest RPKM value was 16.94-fold that of the lowest value (20763.14 for *Gli-α-8* compared with 1225.46 for *Gli-α-10*) (Fig 2A and S6 Table). Compared with the expressed gliadin genes, the RPKM values of the genes with internal stop codons were extremely low, demonstrating that these genes were not expressed and the sequences of all the characterized gliadin genes were correct (S6 Table). In addition, the inability to detect the transcripts of the gliadin genes with intact ORFs in the flag leaves demonstrated the endosperm-specific expression of the gliadin genes in wheat (Fig 2A and S6 Table). To confirm the RNA-Seq data for the expressed genes, a qRT-PCR analysis was performed with nine α- and γ-gliadin genes, and their relative expression patterns across the grain filling stages were consistent with those observed using RNA-Seq, demonstrating the accuracy of the RNA-Seq data (Fig 2).

### Assignment of gliadin genes to their corresponding protein products via proteomic analysis

To characterize the gliadin genes in the aforementioned experiments and dynamic expression pattern analysis, the native protein products encoded by the active gliadin genes had to be identified. Thus, the gliadin extract of the seed storage proteins from PI428198 were separated by 2-DE, and then the mass spectra and peptide mass fingerprints (PMFs) of the excised protein spots were analyzed using MALDI-TOF/TOF-MS (AB SCIEX 5800).

**Fig 3:**
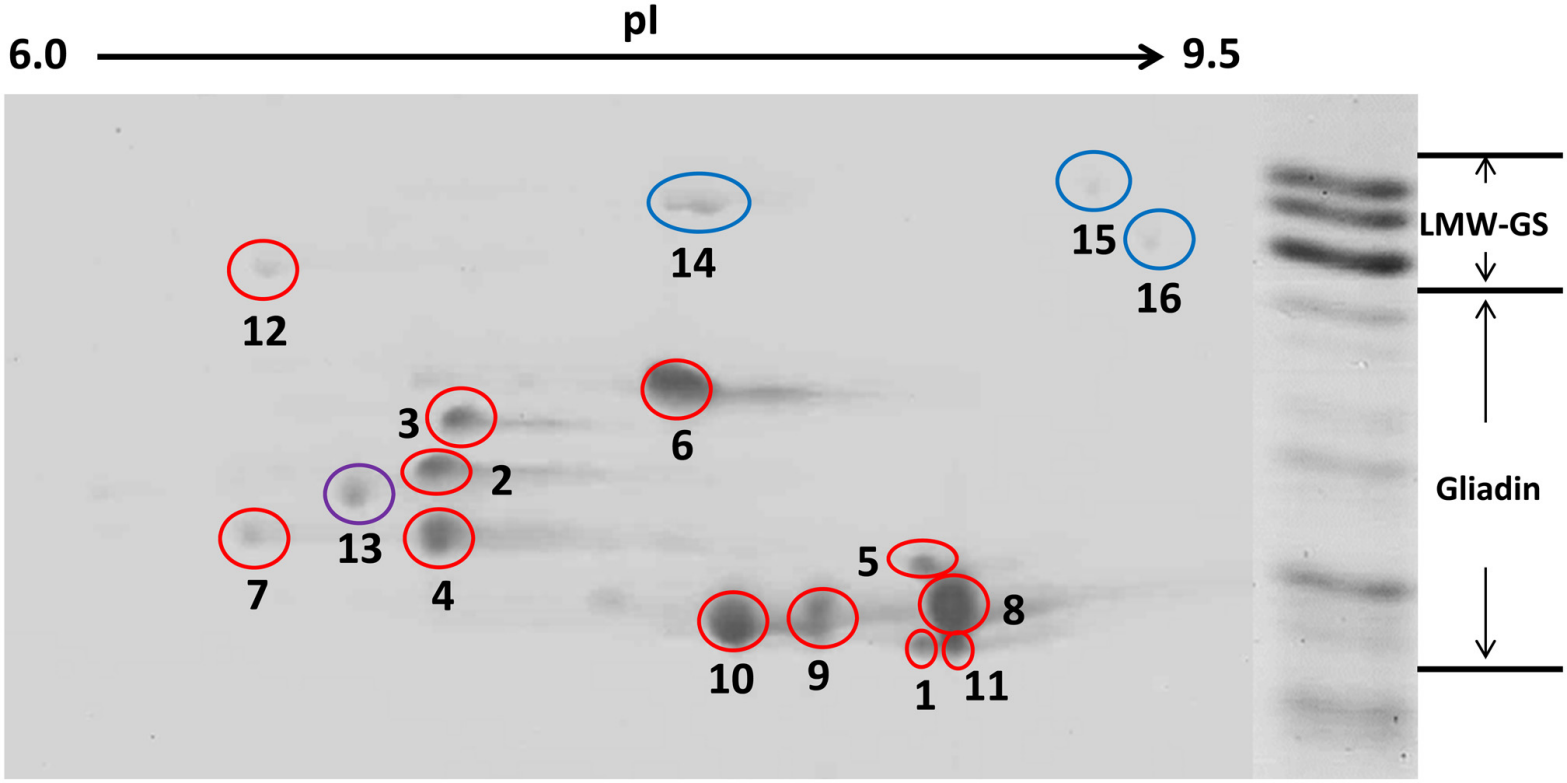
Identification of the gliadin protein spots from *T*. *urartu* after resolution with 2-DE. Gliadins were prepared from mature grains, separated by 2-DE, and further identified via MALDI-TOF/TOF-MS analysis. Shown on the right side is the SDS-PAGE separation of prolamins from *T*. *urartu*. The high-molecular-weight glutenin subunit protein spots are not shown because of limited space. The spots in red circles are gliadins, the spots in blue circles are LMW-GSs (spot 14, KM085281, MW: 38.06, pI: 7.91; spot 15, KM085304, MW: 38.56 pI: 8.5; spot 16, KM085275, MW: 37.52, pI: 8.71), and the spot in purple is avenin-3 (TRIUR3_09156, MW: 35.1, pI: 7.66).

In the 2-DE gel, 16 major spots in the regions of the gliadins and low-molecular-weight glutenin subunit (LMW-GS) genes were marked (Fig 3), excised, and analyzed. After performing the bioinformatics analysis of the PMFs and MALDI-TOF/TOF-MS spectra, 12 of the spots were found to be gliadin proteins (red circles) (Fig 3, Table 2, S7 and S8 Tables). The MALDI-TOF/TOF-MS spectra obtained from the protein spots were carefully compared with the predicted amino acid sequences of the 16 active gliadin genes isolated in this work. All of the gliadin spots were precisely matched to the genes cloned above (Table 2 and S7 Table). Of the remaining 12 gliadin spots, seven (spots 1, 2, 3, 4, 5, 6, and 7) were α-gliadins and five (spots 8, 9, 10, 11 and 12) were γ-gliadins; none was ω-gliadin (Fig 3 and Table 2). Among the matched α-gliadin spots, spots 2, 4, 5 and 7 were assigned as the products of *Gli-α-4*, *Gli-α-8*, *Gli-α-9* and *Gli-13*, respectively, with between 3 and 12 peptides per spot matched to the corresponding gliadin (Fig 3 and S7 Table), whereas the remaining three spots (1, 3 and 6) were found to match the predicted proteins of more than one gliadin gene (Fig 3 and S7 Table). This phenomenon might have been caused by the extremely high similarity between these gliadin proteins, which had similar predicted molecular weights and pIs (Fig 1A and Table 2). Spot 1 contained six peptides that corresponded to both Gli-α-2 and Gli-α-3 and one unique peptide (QPQQLPQFEEIRN) to Gli-α-2 (S7 Table), and all six of the identified peptides in spot 6 could match both Gli-α-10 and Gli-α-11. However, spot 3 could represent a mixture of Gli-α-5, Gli-α-6, Gli-α-7 and Gli-α-12; most of its 11 peptides matched all four of these gliadins, which have similar pIs and molecular weights and share high levels of sequence identity (>98%) (Fig 1A and S7 Table). In addition, spots 8, 9 and 10 were assigned to the predicted Gli-γ-1 protein. This phenomenon has been observed in glutenin subunits previously, but the underlying reasons are still unclear [65]. Furthermore, none of the 16 analyzed spots corresponded to the hypothetical polypeptides encoded by the pseudogenes, which were predicted by ignoring the internal stop codons and frameshift mutations (Tables 1 and 2). Except 12 gliadin protein spots, the remaining four spots in the 2-DE gel were three LMW-GSs (spots 14, 15 and 16; blue circles) and one avenin-3 protein (spot 13; purple) (Fig 3, S6 and S7 Tables). In the avenin-3 protein, presence of even number of cysteine residue lead to the formation of intra-chain disulphide bonds, resulting in the monomers in the 2-DE gels (Fig 3), while only traces of three LMW-GSs were detected as monomers due probably to the presence of six intra-chain disulphide bonds (S7 and S8 Tables) or a cross contamination of gliadin by LMW-GS for the chemincal procedure fractionation [55].

**Table 2:** *T*. *urartu* gliadin genes and their corresponding protein spots as identified using 2-DE and MALDI-TOF/TOF-MS.

**Spot**	**%Vol** ^a^	**Gene**	**MW(kD)**	**pI**
**1**	1.98	*Gli-α-2*	32.11	8.27
		*Gli-α-3*	31.63	8.27
**2**	5.09	*Gli-α-4*	34.75	8.49
**3**	4.71	*Gli-α-5*	32.79	7.71
		*Gli-α-6*	33.53	7.71
		*Gli-α-7*	33.92	7.71
		*Gli-α-12*	33.80	7.72
**4**	12.00	*Gli-α-8*	32.62	7.31
**5**	2.21	*Gli-α-9*	33.06	6.69
**6**	18.79	*Gli-α-10*	34.94	8.02
		*Gli-α-11*	34.70	8.02
**7**	1.84	*Gli-α-13*	33.05	6.90
**8**	26.80	*Gli-γ-1*	32.67	8.33
**9**	10.86	*Gli-γ-1*	32.67	8.33
**10**	11.61	*Gli-γ-1*	32.67	8.33
**11**	3.54	*Gli-γ-2*	31.81	8.33
**12**	0.58	*Gli-γ-3*	39.12	7.60

^a^Percentage of the total spot volume of the extracted gliadin fraction that is accounted for by the indicated spot.

The expression levels of all of the active gliadin genes were quantified using ImageMaster platinum 6.0 (GE Healthcare, Little Chalfont, UK) (Table 2). The percent of the total volume accounted for by each individual spot in the extracted gliadin fractions showed that the γ-gliadin Gli-γ-1 was the most abundant gliadin protein in *T*. *urartu*, occupying 49.27% (spot 8 accounted for 26.80%, spot 9 for 10.86%, and spot 10 for 11.61%) of the total extracted gliadin protein in mature seeds; this result is consistent with its high RPKM values in developing grains (Fig 3 and Table 2). Another γ-gliadin, Gli-γ-3 (spot 12), was the least abundant protein and accounted for 0.58% of the total gliadin protein, which is inconsistent with the expression levels of its corresponding gene during grain development (Table 2). This phenomenon was confirmed by the poor correlation between the percent volume of each gliadin and its transcription levels (RPKM values at each stage and total RPKM value across all stages) (Table 2 and S6 Table). In *T*. *urartu* seeds, the γ-gliadins account for most of the gliadin fractions, and with the volume of Gli-γ-2 (spot 11, 3.54%), they accounted for 53.39% of the extracted gliadin proteins (Table 2). Because ω-gliadin spots were not identified, the remaining 46.61% of the gliadins in *T*. *urartu* seeds were α-gliadins. The protein in spot 6, which was encoded by *Gli-α-10* and *Gli-α-11*, accounted for the highest percent volume of the α-gliadins (18.79%), whereas spot 7 (*Gli-α-13*) accounted for only 1.84% of the volume (Fig 3 and Table 2).

### Phylogenetic analysis of the α-gliadin genes

**Fig 4:**
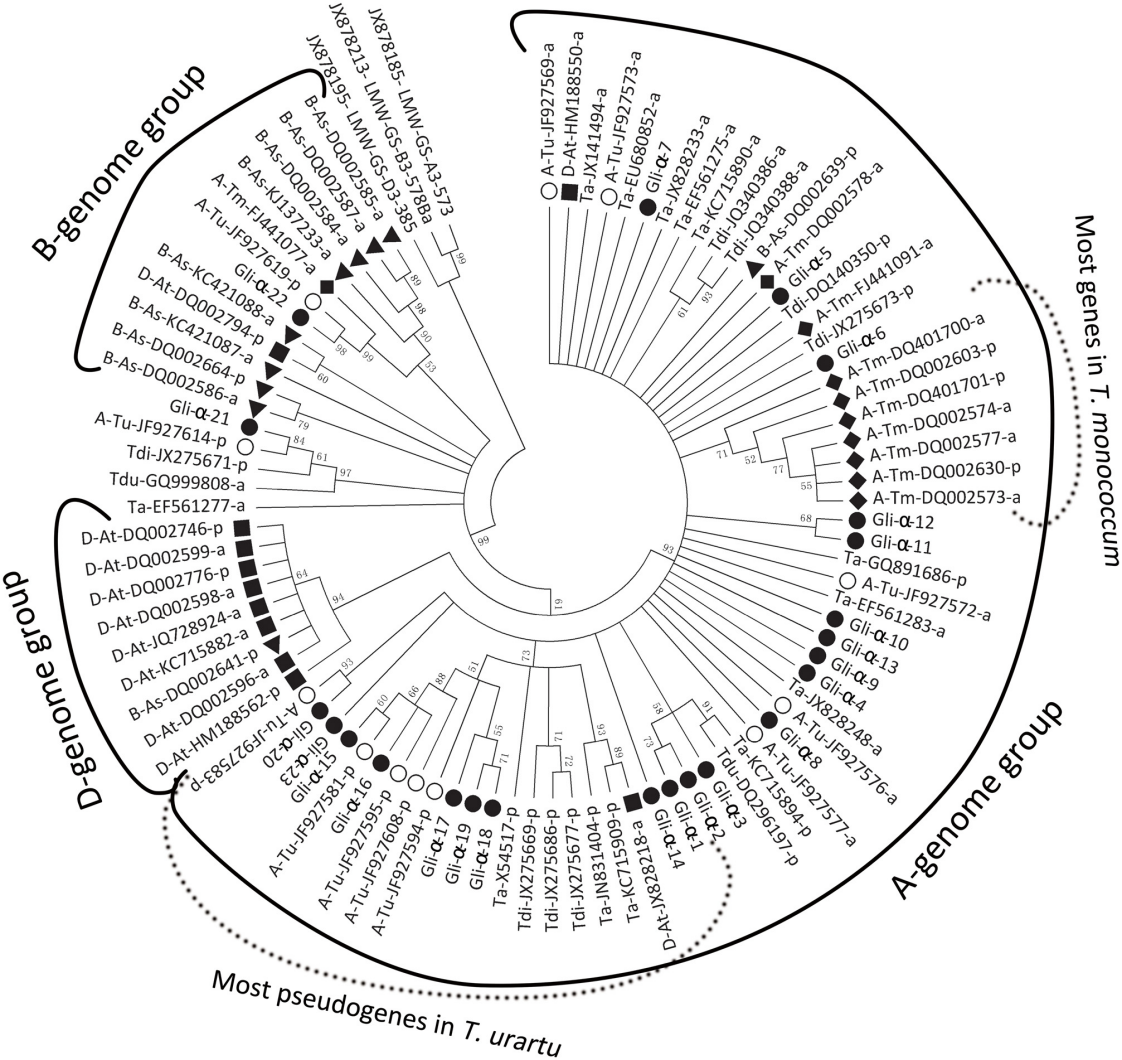
Neighbor-joining tree of the 23 newly identified α-gliadin genes (solid circle) in *T*. *urartu* accession PI428198 and additional α-gliadin genes from *Triticum* and *Aegilops* species. The prefix abbreviations and geometric figures of GenBank accession numbers indicate the different genomes. As: *Ae*. *speltoides*; At: *Ae*. *tauchii*; Ta: *T*. *aestivum*; Tdi: *T*. *dicoccoides*; Tdu: *T*. *durum*; Tm: *T*. *monococcum*. A: A genome; B: B genome; D: D genome. The suffix letters of each accession number, a and p, respresent active and pseudo- genes, respectively.

In the present study, 23 α-gliadin genes from *T*. *urartu*, the A-genome donor of tetraploid and hexaploid wheat, were characterized, and the results facilitated the phylogenetic and evolutionary analysis of this gene family. Based on predicted protein sequences, the phylogenetic relationships among these characterized α-gliadin genes and their orthologs in other *T*. *urartu* accessions and A-, B- and D-genomes from related species in the GenBank were investigated. The phylogenetic tree revealed that all of the α-gliadin genes clustered into three distinct groups (Fig 4). Most of the genes from *T*. *urartu* and *T*. *monococcum* and their homologs in polyploid wheat (*T*. *durum*, *T*. *dicoccoides* and *T*. *aestivum*) were in the A-genome group, and the majority of the genes from *Ae*. *speltoides* and *Ae*. *tauschii* were in the B- and D-genome groups, respectively (Fig 4), which is consistent with the hypothesis that α-gliadin gene family expansion occurred after the ancestors separated into the three *Triticum* genomes [34]. In the A-genome group, the majority of the α-gliadin genes (except DQ002578) in *T*. *monococcum* were assigned into a subgroup, which indicated that the *Gli-2* loci in *T*. *monococcum* might have changed considerably from these in *T*. *urartu* and other related polyploidy wheat after the divergence of the A genome (Fig 4 and S9 Table). Almost all of the α-gliadin genes characterized in *T*. *urartu* had homologs in tetraploid and hexaploid wheat (e.g., *Gli-α-15~19* with X54517 in *T*. *aestivum*, and *Gli-α-21* with GQ999808 and JX275671 in *T*. *durum and in T*. *dicoccoides*, respectively), and this result is not unexpected for *T*. *urartu* is the A-genome donor of polyploid wheat species (Fig 4 and S9 Table) [40].

Interestingly, *Gli-α-22* and JF927619 in *T*. *urartu* and FJ441077 in *T*. *monococcum* included homologs in the B-genome, and DQ002639 from *Ae*. *speltoides* was also assigned to the A-genome group (Fig 4), implying that these genes originated before the divergence of the A- and B-genomes and supporting the hypothesis that the A- and B-genomes diverged from a common ancestor [66]. Moreover, two (HM188550 and JX828218) and one (DQ002794) α-gliadin genes from *Ae*. *tauchii* were distributed in the A- and B- genome groups, respectively. This phenomenon was consistent with the fact that D genome is the homoploid hybrid of the A- and B-genomes [66]. Remarkably, most of the *T*. *urartu* α-gliadin genes, both pseudo and functional, were clustered into two subgroups (Fig 4), with the pseudogenes containing lower sequence identities (>82%) and increased variability in the number of cysteines (5–7) compared with these of the functional genes (>95% and six cysteines) (Fig 1A). These data demonstrated that the pseudogenes and functional genes experienced two distinct evolutionary mechanisms, with the functional genes remaining more conserved during the evolutionary process [67]. The phylogenetic analysis also assigned *Gli-α-21* to a unique clade that was closer to the root than the A and D gliadin gene groups, suggesting that *Gli-α-21* might be an ancient gliadin gene. Additionally, *Gli-α-21*’s homologs were present in *T*. *durum*, *T*. *dicoccoides* and *T*. *aestivum*, suggesting its conservation during wheat evolution.

## Discussion

### Full complement of *T*. *urartu* gliadin genes

Numerous gliadin genes have been characterized using genomic DNA, EST and BAC libraries from common wheat and its relatives [25–27, 31]. With the conserved PCR primers, 701 α-gliadin genes were collected from 29 diploid *Triticeae* species [31]. However, the primers used for PCR amplification and probes employed in BAC library screenings were generally designed from previously identified gene sequences, which would make it difficult to clone new genes that have different sequences at the priming sites [31]. The most comprehensive method of determining the entire composition of a gene family is to utilize genomic data [68]. Nevertheless, the available *Triticum* genome data are not sufficient to predict all of the members of this complex gene family because of inaccuracies in the assembly of repetitive sequences, which account for a large proportion of the genome [41, 69]. In the present work, a PCR cloning strategy was used in combination with gene predictions from genomic data to identify the full complement of the gliadin family genes in *T*. *urartu*, and this strategy resulted in the characterization of 28 gliadin genes in *T*. *urartu*. To the best of our knowledge, this is the largest number of genes identified in a single germplasm. Of these gliadin genes, only nine were identified from genome sequencing data, suggesting that predicting genes using genomic data that contain high proportions of repetitive sequences is difficult (Table 1).

Among the gliadin genes, the α-type had 23 copies, which was lower than the estimated copy number (25–150) in individual haploid genomes [10, 70]. However, the percentage of active genes found in this work (47.83%) was much higher than that found in previous studies (5%-28%) [10, 31, 32, 34]. α-gliadins play a vital role in determining dough quality, eg. the overall dough strength (mixing time, mixing stability and work input) and the mixing tolerance (resistance breakdown and peak dough resistance values) of the mixograph properties resulting in the change of loaf volume [13], and the extensibility of the dough and gluten matrix improving the diameter and overall quality of the tortillas (http://www.ncbi.nlm.nih.gov/pubmed/19170634/). Thus, *T*. *urartu* could be an excellent genetic resource for wheat breeding because of its larger number of protein-coding α-gliadin genes. Although the copy numbers of γ-type (three) and ω-type (two) gliadin genes were lower than those of previously identified genes in *T*. *urartu* (14 γ- and two ω-gliadin genes) [35, 45], all three of the *T*. *urartu* γ-gliadin genes are protein-coding genes, which is consistent with the high proportion (86%) of active genes in common wheat [35], and all of the protein spots in the 2-DE gel were assigned to the cloned genes. Of the 28 cloned gliadin genes, approximately 50% were pseudogenes caused primarily by mutations that converted glutamine codons (CAA and CAG) to stop codons (TAA and TAG); such mutations are more likely in gluten protein genes because of their high glutamine content (Fig 1).

### Gliadin genes of *T*. *urartu*

All of the gliadin gene sequences from *T*. *urartu* were analyzed using the NCBI nucleotide BLAST program. Almost all of the genes had homologs with a high level of sequence identity (93%-99%) with wheat and/or related species, which is consistent with *T*. *urartu* being the A-genome donor of polyploid wheat species (S9 Table) [40]. Interestingly, *Gli-ω-1* had no homologs in the NCBI database, suggesting that it might be a new ω-gliadin variant.

As a unique feature of gliadin genes, six cysteines were generally characterized in most of the α-gliadins in diploid *Triticeae* species with a few exceptions in non-A genome species [31]. In this work, though most of the α-gliadins had six cysteines, Gli-α-17, Gli-α-18 and Gli-α-19 contained an additional cysteine in the UI domain, which enables them to participate in gluten polymerization through intermolecular bonds with other storage proteins (Fig 1A) [59]. All of the gliadins identified in this work contain toxic epitopes associated with CD [22, 58, 61]. Based on the distributions of their CD-toxic epitopes and structural variation within the QI and QII domains, the α-gliadin genes can be divided into three groups: group I (containing a Q-to-A point mutation in QI and the DQ8-glia-α1 epitope), group II (containing a Q-to-K point mutation in QII and the DQ2.5-glia-α1a and DQ2.5-glia-α3 epitopes) and group III (containing no point mutations and 3–4 epitopes) [71]. Based on these criteria, Gli-α-21 and Gli-α-22 belong to group I and Gli-α-4 to Gli-α-13 and Gli-α-18 belong to group II (Fig 1A). However, the remaining ten α-gliadin genes could not be placed into any of these groups because they contained either one glia-α9 epitope or one glia-α20 epitope, which is an entirely new distribution of toxic epitopes (Fig 1A). The chromosomal location of the α-gliadin genes can be determined by their chromosome-specific toxic epitopes; in common wheat, α-gliadin genes derived from chromosome 6A almost invariably contain DQ2.5-glia-α1a and DQ2.5-glia-α3 rather than DQ8-glia-α1 and DQ2.5-glia-α2 [34]. However, both Gli-α-21 and Gli-α-22 from *T*. *urartu* are on chromosome 6A and contain DQ8-glia-α1 (Fig 1A). Based on the first three amino acids of their mature proteins, ω-gliadins are classified into ARQ/E, KEL, SRL and TRQ types [45]. Although Gli-ω-2 contained a Q-to-H point mutation in the third amino acid of its mature protein, its typical 4:3:1 Q:P:F ratio suggests that it might be a new variant of the ARQ/E type (Fig 1C).

### Expression pattern of the gliadin genes

Proteomic methods, including sodium dodecyl sulfate polyacrylamide gel electrophoresis (SDS-PAGE), size-exclusion and reversed-phase high-performance liquid chromatography (HPLC) and matrix-assisted laser desorption/ionization time-of-flight mass spectrometry (MALDI/TOF-MS), and qRT-PCR analyses have suggested that gliadin synthesis in common wheat begins at 5–8 DPA [14, 47–49]. Similarly, the RNA-Seq analysis of all of the gliadin genes studied in this work showed that their transcription began during the same period, with their RPKMs hardly detectable at 5 DPA and clear at 10 DPA (Fig 2). Once transcription started, all of the gliadin genes studied in this work displayed the same expression profile, with maximum expression at 15 DPA and a gradual decline through 20 and 25 DPAs (Fig 2). This expression pattern corroborated the findings of qRT-PCR-based transcriptomic analyses of α- and γ-gliadin genes in common wheat [47, 72]. However, an EST analysis showed two distinct expression patterns for the α- and β-gliadin genes in common wheat, and other complex regulatory mechanisms might influence the expression of genes, excluding those containing a prolamin box [9, 73]. Certain prolamin genes with premature stop codons are transcribed during grain development, and they are controlled transcriptionally and/or post-transcriptionally [74]. Nevertheless, the RPKM values of all the pseudogenes studied in this work were extremely low, which indicated that they were silenced (S6 Table). The expression of γ-gliadin genes is up to 10-fold higher than that of the other gliadin family members, which has been shown by qRT-PCR [72]. In the present study, two of the three γ-gliadin genes, namely *Gli-γ-1* and *Gli-γ-3*, had higher RPKM values than did the α- and ω-type genes, and Gli-γ-1 accounted for 49.27% of the total gliadin protein in mature seeds. However, despite its high RPKM values, Gli-γ-3 only accounted for 0.58% of the total gliadin protein, suggesting that this gene might be regulated at the translational level (Fig 2A and Table 2).

In summary, this study performed a comprehensive analysis of the complement of gliadin genes in *T*. *urartu* by combining a PCR-based cloning strategy that analyzed genomic DNA and mRNA in developing seeds with a gene prediction method based on genomic sequence data. The expression patterns of these gliadin genes and accumulation of encoded proteins were also elucidated using RNA-Seq and a combined 2-DE and MALDI-TOF/TOF-MS analysis, respectively. Collectively, this study has provided important insights into the composition and expression pattern of the gliadin gene families in *T*. *urartu*, and these data can be used to better understand the gene families in *Triticeae* and improve the quality of common wheat.

## Supporting Information

S1 Fig Sequence alignments of the *T*. *urartu* α-gliadin genes. (TIF)

S1 Table Conserved primers used for cloning the gliadin genes from *T*. *urartu*. (DOCX)

S2 Table Query sequences used in the basic local alignment to search gliadin genes in the PI428198 genome sequence database. (DOCX)

S3 Table Gene-specific qRT-PCR primers. (DOCX)

S4 Table Toxic epitope content of the α-gliadins in *T*. *urartu* accession PI428198. (DOCX)

S5 Table Global statistics of the RNA-Seq data from grain samples of *T*. *urartu* accession PI428198 at five developmental stages. (DOCX)

S6 Table The RPKM values of each gliadin gene as calculated from RNA-Seq data and normalized expression levels of the nine genes analyzed via RT-PCR from *T*. *urartu* accession PI428198 grain at five developmental stages. (DOCX)

S7 Table MALDI-TOF/TOF-MS identification of the protein spots from the gliadin fractions of *T*. *urartu* accession PI428198 after separation via 2-DE. (DOCX)

S8 Table LC-MS/MS identification of the protein spots from the LMW-GS fractions of *T*. *urartu* accession PI428198 after separation via 2-DE. (DOCX)

S9 Table The level of nucleotide sequence identity between the gliadin genes of *T*. *urartu* accession PI428198 and previously reported genes/allelic variants. (DOCX)
