# Supplementary material for: Genome-, Transcriptome- and Proteome-Wide Analyses of the Gliadin Gene Families in Triticum urartu
Source: PLoS One. 2015 Jul 1;10(7):e0131559. doi: 10.1371/journal.pone.0131559 (PMC4489009; doi:10.1371/journal.pone.0131559)
Supplement: S1 Table — (DOCX) [file pone.0131559.s002.docx]

**S1 Table. Conserved primers used for cloning the gliadin genes from *T. urartu*.**

| **Group** | **Primer** | **Sequence (5'-3')** |
| --- | --- | --- |
| **alpha-gliadin** | *alpha-F1* | ATGAAGACCTTTCTCATCC |
|  | *alpha-R1* | GTTAGTACCGAAGATGCC |
|  | *alpha-F2* | ATGGTTAGAGTTCCAGTGCCA |
|  | *alpha-R2* | TCATCGATAGTTAGTACCGAA |
|  | *alpha/beta-F1* | ATGAAGACCTTTCTCATCCTTG |
|  | *alpha/beta-R1* | CAGTTGGTACCGAAGATGC |
| **gamma-gliadin** | *gamma-F1* | TATTAGTTAACGCAAATCCAC(C/T)ATG |
|  | *gamma-R1* | GATGAATCAGCTAAGCAACGATG |
|  | *gamma-F2* | CTTCACACAACTAGAGCACAAG |
|  | *gamma-R2* | TCGTTACATCTATTGGTGCATCAG |
|  | *gamma-F3* | ATGAAGACCTTACTCATCC |
|  | *gamma-R3* | GGACAWAGACRTTGCACATG |
| **omega-gliadin** | *omega-F1* | ATGAAGACCTTCCTCATCTTTG |
|  | *omega-R1* | TCATTGGCCACCGATGCTTGT |
