# Supplementary material for: Genome-, Transcriptome- and Proteome-Wide Analyses of the Gliadin Gene Families in Triticum urartu
Source: PLoS One. 2015 Jul 1;10(7):e0131559. doi: 10.1371/journal.pone.0131559 (PMC4489009; doi:10.1371/journal.pone.0131559)
Supplement: S2 Table — (DOCX) [file pone.0131559.s003.docx]

**S2 Table. Query sequences used in the basic local alignment to search gliadin genes in the PI428198 genome sequence database.**

| **GenBank Accession** | **Organism** | |  | | **GenBank Accession** | | **Organism** | |  | | **GenBank Accession** | | **Organism** | |
| --- | --- | --- | --- | --- | --- | --- | --- | --- | --- | --- | --- | --- | --- | --- |
| α-gliadin |  |  | | γ-gliadin | |  | |  | | Hordein | |  | |  |
| DQ002585 | *Aegilops speltoides* |  | | FJ006702 | | *Aegilops speltoides* | |  | | AY338365 | | *Hordeum chilense* | |  |
| DQ002639 | *Aegilops speltoides* |  | | JQ269764 | | *Aegilops speltoides* | |  | | AY338366 | | *Hordeum chilense* | |  |
| DQ002664 | *Aegilops speltoides* |  | | JQ269777 | | *Aegilops speltoides* | |  | | AY338367 | | *Hordeum chilense* | |  |
| KC421087 | *Aegilops speltoides* |  | | JQ269787 | | *Aegilops speltoides* | |  | | AY338368 | | *Hordeum chilense* | |  |
| DQ002596 | *Aegilops tauschii* |  | | JQ269802 | | *Aegilops tauschii* | |  | | AY338369 | | *Hordeum chilense* | |  |
| HM188550 | *Aegilops tauschii* |  | | KF880538 | | *Aegilops tauschii* | |  | | AY338370 | | *Hordeum chilense* | |  |
| HM188562 | *Aegilops tauschii* |  | | KF880550 | | *Aegilops tauschii* | |  | | AY338371 | | *Hordeum chilense* | |  |
| EF561277 | *Triticum aestivum* |  | | KF880577 | | *Aegilops tauschii* | |  | | AY338372 | | *Hordeum chilense* | |  |
| EU680852 | *Triticum aestivum* |  | | KF880580 | | *Aegilops tauschii* | |  | | AY338373 | | *Hordeum chilense* | |  |
| GQ891686 | *Triticum aestivum* |  | | FJ006593 | | *Triticum aestivum* | |  | | AY338374 | | *Hordeum chilense* | |  |
| JX828248 | *Triticum aestivum* |  | | FJ006602 | | *Triticum aestivum* | |  | | AY338375 | | *Hordeum chilense* | |  |
| KC715890 | *Triticum aestivum* |  | | FJ006615 | | *Triticum aestivum* | |  | | AY338376 | | *Hordeum chilense* | |  |
| DQ140350 | *Triticum dicoccoides* |  | | FJ231103 | | *Triticum aestivum* | |  | | AY338377 | | *Hordeum chilense* | |  |
| JQ340386 | *Triticum dicoccoides* |  | | KC715955 | | *Triticum aestivum* | |  | | AY338378 | | *Hordeum chilense* | |  |
| JX275669 | *Triticum dicoccoides* |  | | KC715968 | | *Triticum aestivum* | |  | | AY338379 | | *Hordeum chilense* | |  |
| DQ296197 | *Triticum durum* |  | | KC715969 | | *Triticum aestivum* | |  | | AY338381 | | *Hordeum chilense* | |  |
| GQ999808 | *Triticum durum* |  | | KC715997 | | *Triticum aestivum* | |  | | AY338384 | | *Hordeum chilense* | |  |
| DQ002578 | *Triticum monococcum* |  | | KF412606 | | *Triticum aestivum* | |  | | AY338385 | | *Hordeum chilense* | |  |
| DQ401700 | *Triticum monococcum* |  | | KF412608 | | *Triticum aestivum* | |  | | AJ580585 | | *Hordeum vulgare* | |  |
| JF927569 | *Triticum urartu* |  | | FJ006563 | | *Triticum dicoccoides* | |  | | JQ867078 | | *Hordeum vulgare* | |  |
| JF927595 | *Triticum urartu* |  | | FJ006576 | | *Triticum dicoccoides* | |  | | JQ867079 | | *Hordeum vulgare* | |  |
| JF927614 | *Triticum urartu* |  | | FJ006583 | | *Triticum durum* | |  | | X72628 | | *Hordeum vulgare* | |  |
|  |  |  | | FJ006584 | | *Triticum durum* | |  | |  | |  | |  |
| ω-gliadin |  |  | | FJ006586 | | *Triticum durum* | |  | | avenin-like | |  | |  |
| DQ307378 | *Triticum aestivum* |  | | FJ006657 | | *Triticum monococcum* | |  | | AF469489 | | *Triticum aestivum* | |  |
| FJ598070 | *Triticum aestivum* |  | | FJ441094 | | *Triticum monococcum* | |  | | EF526509 | | *Triticum aestivum* | |  |
| KC716069 | *Triticum aestivum* |  | | JQ269816 | | *Triticum monococcum* | |  | | GQ903578 | | *Triticum aestivum* | |  |
| KC716080 | *Triticum aestivum* |  | | JX081266 | | *Triticum monococcum* | |  | | GQ903579 | | *Triticum aestivum* | |  |
| KF412597 | *Triticum aestivum* |  | | FJ006634 | | *Triticum urartu* | |  | | HM027635 | | *Triticum aestivum* | |  |
| KF412598 | *Triticum aestivum* |  | | FJ006640 | | *Triticum urartu* | |  | | JN542446 | | *Triticum aestivum* | |  |
| KM879356 | *Triticum aestivum* |  | | FJ006662 | | *Triticum urartu* | |  | | JN542449 | | *Triticum aestivum* | |  |
| DQ317535 | *Triticum dicoccoides* |  | | FJ006665 | | *Triticum urartu* | |  | | JN542450 | | *Triticum aestivum* | |  |
| GU220053 | *Triticum dicoccoides* |  | | secaline | |  | |  | | JN542453 | | *Triticum aestivum* | |  |
| GU220054 | *Triticum dicoccoides* |  | | AF000227 | | *Secale cereale* | |  | | JN542456 | | *Triticum aestivum* | |  |
| HM016497 | *Triticum monococcum* |  | | AJ862830 | | *Secale cereale* | |  | | JN542460 | | *Triticum aestivum* | |  |
| HM016498 | *Triticum monococcum* |  | | AJ862831 | | *Secale cereale* | |  | | JN542462 | | *Triticum aestivum* | |  |
| HM016507 | *Triticum monococcum* |  | | EU368041 | | *Secale cereale* | |  | | KF840202 | | *Triticum aestivum* | |  |
| KJ789864 | *Triticum monococcum* |  | | FN555204 | | *Secale cereale* | |  | | KF840204 | | *Triticum aestivum* | |  |
| AY667097 | *Triticum urartu* |  | | HE858611 | | *Secale cereale* | |  | |  | |  | |  |
| JN630581 | *Triticum urartu* |  | | HE858614 | | *Secale cereale* | |  | |  | |  | |  |
