# Supplementary material for: Genome-, Transcriptome- and Proteome-Wide Analyses of the Gliadin Gene Families in Triticum urartu
Source: PLoS One. 2015 Jul 1;10(7):e0131559. doi: 10.1371/journal.pone.0131559 (PMC4489009; doi:10.1371/journal.pone.0131559)
Supplement: S3 Table — (DOCX) [file pone.0131559.s004.docx]

**S3 Table. Gene-specific qRT-PCR primers.**

| **Primer** | **Sequence (5'-3')** |
| --- | --- |
| ***Gli-α-2-F*** | CATCTCAGCAACAGCCTCG |
| ***Gli-α-2-R*** | TGCGGAAATAGTTGCAGCTGT |
| ***Gli-α-3-F*** | GAAGATCACAAGTTTTGCAACATAC |
| ***Gli-α-3-R*** | TTGTTGATGCAGAATAATAGCATGT |
| ***Gli-α-4-F*** | CAACAGCCACAAGAGCAAGATA |
| ***Gli-α-4-R*** | TGGTTGTTGTGGTCGAAATG |
| ***Gli-α-5-F*** | ACTGCAGTTAGAGTTCCAGTTCT |
| ***Gli-α-5-R*** | GTTGTGATGGAAATGGTTGC |
| ***Gli-α-6-F*** | AGCCACAAGAGCAAGTTCCTTC |
| ***Gli-α-6-R*** | GTTGCGGTTGTGGATATGGT |
| ***Gli-α-9-F*** | ACAACAAGAACAACAAATCCTTGAT |
| ***Gli-α-9-R*** | GCGACTGCTCAGGGATCT |
| ***Gli-α-12-F*** | AACAACAACAACAACAACCAGCA |
| ***Gli-α-12-R*** | GCAAATTGCAGGTAGCGTCT |
| ***Gli-γ-1-F*** | GAAGTGATTGCCAGGTGTTG |
| ***Gli-γ-1-R*** | TTGGATGATGCCCTGACCT |
| ***Gli-γ-2-F*** | GAAGTGATTGCCAGGTGTTC |
| ***Gli-γ-2-R*** | TTGGATGATGCCCTGACCT |
