# Supplementary material for: Genome-, Transcriptome- and Proteome-Wide Analyses of the Gliadin Gene Families in Triticum urartu
Source: PLoS One. 2015 Jul 1;10(7):e0131559. doi: 10.1371/journal.pone.0131559 (PMC4489009; doi:10.1371/journal.pone.0131559)
Supplement: S4 Table — (DOCX) [file pone.0131559.s005.docx]

**S4 Table. Toxic epitope content of the α-gliadins in *T. urartu* accession PI428198.**

|  | **12-residue peptide** | **19-residue peptide** | **DQ8-glia-α1** | **DQ2.5-glia-α1a** | **DQ2.5-glia-α3** |
| --- | --- | --- | --- | --- | --- |
| **Gli-α-1** | 0 | 1 | 0 | 0 | 1 |
| **Gli-α-2** | 0 | 1 | 0 | 0 | 1 |
| **Gli-α-3** | 0 | 0 | 0 | 0 | 1 |
| **Gli-α-4** | 1 | 1 | 0 | 1 | 1 |
| **Gli-α-5** | 1 | 1 | 0 | 1 | 1 |
| **Gli-α-6** | 0 | 0 | 0 | 1 | 1 |
| **Gli-α-7** | 1 | 0 | 0 | 1 | 1 |
| **Gli-α-8** | 1 | 1 | 0 | 1 | 1 |
| **Gli-α-9** | 1 | 1 | 0 | 1 | 1 |
| **Gli-α-10** | 1 | 1 | 0 | 1 | 1 |
| **Gli-α-11** | 1 | 1 | 0 | 1 | 1 |
| **Gli-α-12** | 1 | 1 | 0 | 1 | 1 |
| **Gli-α-13** | 1 | 1 | 0 | 1 | 1 |
| **Gli-α-14** | 0 | 1 | 0 | 0 | 1 |
| **Gli-α-15** | 0 | 0 | 0 | 1 | 0 |
| **Gli-α-16** | 0 | 0 | 0 | 1 | 0 |
| **Gli-α-17** | 0 | 0 | 0 | 0 | 1 |
| **Gli-α-18** | 0 | 0 | 0 | 1 | 1 |
| **Gli-α-19** | 0 | 0 | 0 | 0 | 1 |
| **Gli-α-20** | 0 | 0 | 0 | 0 | 1 |
| **Gli-α-21** | 0 | 0 | 1 | 0 | 0 |
| **Gli-α-22** | 0 | 0 | 1 | 0 | 0 |
| **Gli-α-23** | 0 | 0 | 0 | 0 | 1 |
