# Supplementary material for: Genome-, Transcriptome- and Proteome-Wide Analyses of the Gliadin Gene Families in Triticum urartu
Source: PLoS One. 2015 Jul 1;10(7):e0131559. doi: 10.1371/journal.pone.0131559 (PMC4489009; doi:10.1371/journal.pone.0131559)
Supplement: S5 Table — (DOCX) [file pone.0131559.s006.docx]

**S5 Table. Global statistics of the RNA-Seq data from grain samples of *T. urartu* accession PI428198 at five developmental stages.**

| **Sample ID** | **Yield (Mb)** | **% PF** | **Raw reads** | **% of raw clusters per lane** | **% Perfect Index Reads** | **% One Mismatch Reads (Index)** | **% of >= Q30 Bases (PF)** | **Mean Quality Score (PF)** | **Clean reads** |
| --- | --- | --- | --- | --- | --- | --- | --- | --- | --- |
| **10 DPA-FL-1^a^** | 1999 | 100 | 19,985,666 | 4.76 | 100 | 0 | 90.31 | 34.79 | 19,040,632 |
| **10 DPA-FL-2^a^** | 2147 | 100 | 21,474,674 | 5.11 | 100 | 0 | 90.50 | 34.89 | 20,549,334 |
| **10 DPA-FL-3^a^** | 2219 | 100 | 22,185,442 | 5.28 | 100 | 0 | 89.66 | 34.64 | 21,070,616 |
| **5 DPA-1** | 2459 | 100 | 24,590,424 | 5.85 | 100 | 0 | 90.67 | 34.97 | 23,514,328 |
| **5 DPA-2** | 2523 | 100 | 25,234,252 | 6.01 | 100 | 0 | 91.00 | 35.04 | 24,224,106 |
| **5 DPA-3** | 2490 | 100 | 24,900,170 | 5.93 | 100 | 0 | 89.22 | 34.47 | 23,528,896 |
| **10 DPA-1** | 2417 | 100 | 24,165,272 | 5.75 | 100 | 0 | 90.20 | 34.82 | 22,928,974 |
| **10 DPA-2** | 2434 | 100 | 24,339,102 | 5.80 | 100 | 0 | 90.63 | 34.96 | 23,230,402 |
| **10 DPA-3** | 2382 | 100 | 23,816,242 | 5.67 | 100 | 0 | 90.40 | 34.91 | 22,769,334 |
| **15 DPA-1** | 2065 | 100 | 20,650,010 | 4.92 | 100 | 0 | 89.95 | 34.77 | 19,682,506 |
| **15 DPA-2** | 2244 | 100 | 22,439,694 | 5.34 | 100 | 0 | 89.65 | 34.66 | 21,311,874 |
| **15 DPA-3** | 1959 | 100 | 19,594,750 | 4.67 | 100 | 0 | 89.71 | 34.70 | 18,658,744 |
| **20 DPA-1** | 2570 | 100 | 25,698,316 | 6.12 | 100 | 0 | 90.09 | 34.63 | 24,235,186 |
| **20 DPA-2** | 2387 | 100 | 23,870,486 | 5.68 | 100 | 0 | 90.54 | 34.81 | 22,796,822 |
| **20 DPA-3** | 2383 | 100 | 23,826,012 | 5.67 | 100 | 0 | 90.34 | 34.75 | 22,715,382 |
| **25 DPA-1** | 2448 | 100 | 24,483,074 | 5.83 | 100 | 0 | 90.00 | 34.64 | 23,321,156 |
| **25 DPA-2** | 2074 | 100 | 20,738,926 | 4.94 | 100 | 0 | 89.45 | 34.59 | 19,771,806 |
| **25 DPA-3** | 1953 | 100 | 19,527,124 | 4.65 | 100 | 0 | 89.67 | 34.62 | 18,667,590 |

^a^Flag leaves of 10 days post anthesis, and 1, 2 and 3 mean three biological replicates.
