# Supplementary material for: Genome-, Transcriptome- and Proteome-Wide Analyses of the Gliadin Gene Families in Triticum urartu
Source: PLoS One. 2015 Jul 1;10(7):e0131559. doi: 10.1371/journal.pone.0131559 (PMC4489009; doi:10.1371/journal.pone.0131559)
Supplement: S6 Table — (DOCX) [file pone.0131559.s007.docx]

**S6 Table. The RPKM values of each gliadin gene as calculated from RNA-Seq data and normalized expression levels of the nine genes analyzed via RT-PCR from *T. urartu* accession PI428198 grain at five developmental stages.**

| **Gene** | **RPKM of RNA-Seq** | | | | | |  | **Normalized expression level of RT-PCR** | | | | | |
| --- | --- | --- | --- | --- | --- | --- | --- | --- | --- | --- | --- | --- | --- |
|  | **10 DPA-FL** | **5 DPA** | **10 DPA** | **15 DPA** | **20 DPA** | **25 DPA** |  | **10 DPA-FL** | **5 DPA** | **10 DPA** | **15 DPA** | **20 DPA** | **25 DPA** |
| ***Gli-α-1*** | 0 | 0.07 | 518.72 | 590.03 | 100.90 | 106.52 |  |  |  |  |  |  |  |
| ***Gli-α-2*** | 0 | 0.47 | 6013.25 | 11572.06 | 6532.77 | 1480.70 |  | 0 | 0 | 4.05 | 24.86 | 5.81 | 0.63 |
| ***Gli-α-3*** | 1.15 | 0.67 | 12749.32 | 30588.85 | 14864.49 | 4510.20 |  | 0 | 0 | 7.52 | 37.96 | 9.99 | 1.68 |
| ***Gli-α-4*** | 0 | 0.07 | 2256.89 | 2864.64 | 1990.89 | 552.67 |  | 0.02 | 0.01 | 39.58 | 118.74 | 32.26 | 5.56 |
| ***Gli-α-5*** | 0.67 | 1.38 | 14589.26 | 16648.20 | 6703.13 | 2406.94 |  | 0 | 0 | 1.86 | 3.21 | 0.63 | 0.51 |
| ***Gli-α-6*** | 0 | 0.81 | 6023.69 | 8094.28 | 4512.84 | 1772.58 |  | 0.01 | 0 | 6.26 | 16.86 | 6.42 | 2.60 |
| ***Gli-α-7*** | 1.23 | 0.8 | 10560.67 | 19939.89 | 12740.81 | 3880.78 |  |  |  |  |  |  |  |
| ***Gli-α-8*** | 0 | 1.53 | 19951.95 | 39559.93 | 20763.14 | 6729.44 |  |  |  |  |  |  |  |
| ***Gli-α-9*** | 0 | 0.75 | 4462.50 | 5532.49 | 6444.04 | 1387.99 |  | 0 | 0 | 0.29 | 0.71 | 0.07 | 0.13 |
| ***Gli-α-10*** | 0 | 0.07 | 1775.91 | 2699.48 | 1225.46 | 361.86 |  |  |  |  |  |  |  |
| ***Gli-α-11*** | 0 | 0.69 | 6422.75 | 10032.27 | 4568.98 | 1425.03 |  |  |  |  |  |  |  |
| ***Gli-α-12*** | 0.45 | 0.52 | 9990.40 | 16331.71 | 6644.24 | 2170.04 |  | 0 | 0 | 21.02 | 49.88 | 5.69 | 4.24 |
| ***Gli-α-13*** | 0 | 0.41 | 2935.42 | 6085.16 | 2766.87 | 943.74 |  |  |  |  |  |  |  |
| ***Gli-α-14*** | 0 | 0.14 | 189.81 | 263.19 | 71.21 | 33.55 |  |  |  |  |  |  |  |
| ***Gli-α-15*** | 0 | 0.14 | 118.45 | 121.10 | 20.75 | 29.03 |  |  |  |  |  |  |  |
| ***Gli-α-16*** | 0 | 0 | 109.88 | 164.67 | 7.61 | 26.02 |  |  |  |  |  |  |  |
| ***Gli-α-17*** | 0 | 0.07 | 52.66 | 39.65 | 8.99 | 15.46 |  |  |  |  |  |  |  |
| ***Gli-α-18*** | 0 | 0 | 187.69 | 234.95 | 43.62 | 24.67 |  |  |  |  |  |  |  |
| ***Gli-α-19*** | 0 | 0 | 59.38 | 37.49 | 13.80 | 10.56 |  |  |  |  |  |  |  |
| ***Gli-α-20*** | 0 | 0 | 328.43 | 377.43 | 203.90 | 116.96 |  |  |  |  |  |  |  |
| ***Gli-α-21*** | 0 | 0 | 120.40 | 160.89 | 46.32 | 26.37 |  |  |  |  |  |  |  |
| ***Gli-α-22*** | 0 | 0.34 | 380.45 | 785.48 | 415.68 | 317.30 |  |  |  |  |  |  |  |
| ***Gli-α-23*** | 0.26 | 0.07 | 288.95 | 290.72 | 141.80 | 66.83 |  |  |  |  |  |  |  |
| ***Gli-γ-1*** | 2.9 | 5.04 | 45873.05 | 114039.01 | 85446.26 | 23751.22 |  | 0 | 0 | 1.84 | 8.60 | 2.85 | 0.89 |
| ***Gli-γ-2*** | 0.78 | 5.32 | 18087.09 | 24867.71 | 19287.48 | 5708.78 |  | 0.01 | 0 | 1.95 | 6.19 | 3.02 | 0.92 |
| ***Gli-γ-3*** | 1.52 | 3.11 | 43944.41 | 89268.40 | 32038.54 | 11305.05 |  |  |  |  |  |  |  |
| ***Gli-ω-1*** | 3.84 | 3.43 | 30850.63 | 84444.73 | 41230.84 | 21629.78 |  |  |  |  |  |  |  |
| ***Gli-ω-2*** | 0 | 0.13 | 101.38 | 136.52 | 89.23 | 37.87 |  |  |  |  |  |  |  |
