# Supplementary material for: Genome-, Transcriptome- and Proteome-Wide Analyses of the Gliadin Gene Families in Triticum urartu
Source: PLoS One. 2015 Jul 1;10(7):e0131559. doi: 10.1371/journal.pone.0131559 (PMC4489009; doi:10.1371/journal.pone.0131559)
Supplement: S7 Table — (DOCX) [file pone.0131559.s008.docx]

**S7 Table. MALDI-TOF/TOF-MS identification of the protein spots from the gliadin fractions of *T. urartu* accession PI428198 after separation via 2-DE.**

| **Spot** | **Matching gene** | **Mass spectrum** | **Observed** | **Mr(expt)** | **Mr(calc)** | **Ions** | **Predicted gliadin/LMW-GS protein sequence** | **Coverage** |
| --- | --- | --- | --- | --- | --- | --- | --- | --- |
| 1 | *Gli-α-2* | LWQIPEQSQCQA | 1487.73 | 1486.72 | 1486.69 | 3 | MKTFLILALLAIVATTATTAVRVPVPQLQPQNPSQQQPREQVPLVQQQQFLGQQQPFPPQQPYPQPQPFPSQQPYLQLQLFPQPQQPYSQPQPFRPQQPYPQPQPQYSQPQQPISQQQQQQQQQQQQILQQILQQQLIPCMDVVLQQHNIAHGRSQVLQQSTYQLLQGLCCQHLWQIPEQSQCQAIHNVVHAIILHQQQKQQQQPSSQVSFQQPQQQYPSGQGSFRPSQQNPQAQGSVQPQQLPQFEEIRNLALQTLPAMCNVYIPPYCTIAPFGIFGTN | 27.86% |
|  |  | PQQLPQFEEIRNLALQTLPAMCNVYI + Oxidation (M) | 3102.53 | 3101.52 | 3101.57 | 3 |  |  |
|  |  | QLPQFEEIRNL | 1386.68 | 1385.68 | 1385.73 | 37 |  |  |
|  |  | QPQQLPQFEEIRN | 1626.74 | 1625.74 | 1625.82 | 4 |  |  |
|  |  | QQHNIAHGRSQVL | 1487.73 | 1486.72 | 1486.78 | 63 |  |  |
|  |  | RPQQPYPQPQPQ | 1463.68 | 1462.68 | 1462.73 | 50 |  |  |
|  |  | RPQQPYPQPQPQY | 1626.74 | 1625.74 | 1625.80 | 65 |  |  |
|  |  | RPSQQNPQAQGSVQPQQLPQFEEI | 2734.36 | 2733.36 | 2733.35 | 6 |  |  |
|  | *Gli-α-3* | LWQIPEQSQCQA | 1487.73 | 1486.72 | 1486.69 | 3 | MKTFLILALLAVVVTTATTAVRVPVPQLQPQNPSQQQPQEQVPLVQQQQFLGQQQPFPPQQPSPQPQPFPSQQPYLQLQPFPKPQQPYSQPQPFRPQQPYPQPQPQYSQPQQPISQQQQQQQQQILQQILQQQLIPCMDVVLQQHNIAHGRSQVLQQTTYQLLQGLCCQHLWQIPEQSQCQAIHNVVHAIILHQQQKQQQQPSSQVSFQQLQQQYPSGQGSFRPSQQNPQAQGSVLPQQLPQFEEIRNLALQTLPAMCNVYIPPYCTIAPFGIFGTN | 31.77% |
|  |  | PQQLPQFEEIRNLALQTLPAMCNVYI + Oxidation (M) | 3102.53 | 3101.52 | 3101.57 | 3 |  |  |
|  |  | QLPQFEEIRNL | 1386.68 | 1385.68 | 1385.73 | 37 |  |  |
|  |  | QQHNIAHGRSQVL | 1487.73 | 1486.72 | 1486.78 | 63 |  |  |
|  |  | RPQQPYPQPQPQ | 1463.68 | 1462.68 | 1462.73 | 50 |  |  |
|  |  | RPQQPYPQPQPQY | 1626.74 | 1625.74 | 1625.80 | 65 |  |  |
|  |  | VRVPVPQLQPQNPSQQQPQEQVPL | 2734.36 | 2733.36 | 2733.46 | 89 |  |  |
| 2 | *Gli-α-4* | QLPQFEEIRNL | 1386.68 | 1385.68 | 1385.73 | 46 | MKTFLILALLAIVATTATTAVRVPVPQLQPQNPSQQQPQEQVTLVQQQQQFLGQQQPFPPQQPYPQPQPFPSQQPYLQLQPFPQPQLPYSQPQPFRPQQPYPQPQPQYSQPQQPISQQQQQQQQQQQQQQQQQQQQQQKQQPQQQQQILQQILQQQLIPCMDVVLQQHNIAHGRSQVLQQSTYQLLQGLCCQHLWQIPEQSQCQAIHNVVHAIILHQQQKQKQQQPSSQVSFQQPQQQYPLGQGSFRPSQQNPQAQGSVQPHQLPQFEEIRNLALQTLPAMCNVYIPPYCTIAPFGIFGTN | 7.97% |
|  |  | RPQQPYPQPQPQ | 1463.68 | 1462.67 | 1462.73 | 85 |  |  |
|  |  | RPQQPYPQPQPQY | 1626.74 | 1625.73 | 1625.80 | 60 |  |  |
| 3 | *Gli-α-5* | CCQHLW | 903.34 | 902.33 | 902.35 | 31 | MKTFLILALLAIVATTATTAVRVPVLQLQPQNPSQQQPQEQVPLVQQQQFLGQQQPFPPQQPYPQPQPFPSQQPYLQLQPFPQPQLPYSQPQPFRPQQPYPQPQPQYSQPQQPISQQQQQQQQQQQQQQQQQILQQILQQQLIPCMDVVLQQHNIAHGRSQVLQQSTYQLLQELCCQHLWQIPEQSQCQAIHNVVHAIILHQQQKQQQQPSSQVSFQQPLQQYPLGQGSFRPSQQNPQAQGSVQPQQLPQFEEIRNLALQTLPAMCNVYIPPYCTIAPFGIFGTN | 23.86% |
|  |  | IAHGRSQVL | 980.54 | 979.54 | 979.56 | 44 |  |  |
|  |  | LWQIPEQSQCQA | 1487.75 | 1486.74 | 1486.69 | 1 |  |  |
|  |  | NIAHGRSQVL | 1094.58 | 1093.58 | 1093.60 | 72 |  |  |
|  |  | QLPQFEEIRNL | 1386.71 | 1385.70 | 1385.73 | 51 |  |  |
|  |  | QPYPQPQP | 954.46 | 953.45 | 953.46 | 21 |  |  |
|  |  | RPQQPYPQPQPQ | 1463.71 | 1462.70 | 1462.73 | 75 |  |  |
|  |  | RPQQPYPQPQPQY | 1626.77 | 1625.76 | 1625.80 | 75 |  |  |
|  |  | RPSQQNPQ | 954.46 | 953.45 | 953.47 | 58 |  |  |
|  |  | RPSQQNPQAQ | 1153.55 | 1152.54 | 1152.56 | 78 |  |  |
|  | *Gli-α-6* | CCQHLW | 903.34 | 902.33 | 902.35 | 31 | MKTFLILALLAIVATTGTTAVRVPVPQLQPQNPSQQQPQEQVPSVQQQQFLGQEQPFPPQQPYPQPQPFPSQQPYLQLQPFPQPQLPYSQPQPFRPQQPYPQPQPQYSQPQQPISQQQQKQQQQQQQQQQQQQQQEQQILQQILQQQLIPCMDVVLQQHNIAHGRSQVLQQSTYQLLQELCCQHLWQIPEQSQCQAIHNVVHAIILHQQQKQQQQPSSQVSFQQPLQQYPLGRGSFRPSQQNPQAQGSVQPQQLPQFEEIRNLALQTLPAMCNVYIPPYCTIAPFGIFGTN | 23.37% |
|  |  | IAHGRSQVL | 980.54 | 979.54 | 979.56 | 44 |  |  |
|  |  | LWQIPEQSQCQA | 1487.75 | 1486.74 | 1486.69 | 1 |  |  |
|  |  | NIAHGRSQVL | 1094.58 | 1093.58 | 1093.60 | 72 |  |  |
|  |  | QLPQFEEIRNL | 1386.71 | 1385.70 | 1385.73 | 51 |  |  |
|  |  | QPYPQPQP | 954.46 | 953.45 | 953.46 | 21 |  |  |
|  |  | RPQQPYPQPQPQ | 1463.71 | 1462.70 | 1462.73 | 75 |  |  |
|  |  | RPQQPYPQPQPQY | 1626.77 | 1625.76 | 1625.80 | 75 |  |  |
|  |  | RPSQQNPQ | 954.46 | 953.45 | 953.47 | 58 |  |  |
|  |  | RPSQQNPQAQ | 1153.55 | 1152.54 | 1152.56 | 78 |  |  |
|  | *Gli-α-7* | CCQHLW | 903.34 | 902.33 | 902.35 | 31 | MKTFLILALLAIVATTATTAVRVPVPQLQPQNPSQQQPQEQVPLVQQQQFLGQQQPFPPQQSYPQPQPFPSQQPYLQLQPFPQPQLPYSQPQPFRPQQPYPQPQPQYSQPQQPISQQQQQQQQQQQQQQQQQQQQQQQQQQILQQILQQQLIPCMDVVLQQHNIAHGRSQVLQQSTYQLLQELCCQHLWQIPEQSQCQAIHNVVHAIILHQQQKQQQQPSSQVSFQQPLQQYPLGQGSFRPSQQNPQAQGSVQPQQLPQFEEIRNLALQTLPAMCNVYIPPYCTIAPFGIFGTN | 28.57% |
|  |  | IAHGRSQVL | 980.54 | 979.54 | 979.56 | 44 |  |  |
|  |  | LWQIPEQSQCQA | 1487.75 | 1486.74 | 1486.69 | 1 |  |  |
|  |  | NIAHGRSQVL | 1094.58 | 1093.58 | 1093.60 | 72 |  |  |
|  |  | QLPQFEEIRNL | 1386.71 | 1385.70 | 1385.73 | 51 |  |  |
|  |  | QPYPQPQP | 954.46 | 953.45 | 953.46 | 21 |  |  |
|  |  | RPQQPYPQPQPQ | 1463.71 | 1462.70 | 1462.73 | 75 |  |  |
|  |  | RPQQPYPQPQPQY | 1626.77 | 1625.76 | 1625.80 | 75 |  |  |
|  |  | RPSQQNPQ | 954.46 | 953.45 | 953.47 | 58 |  |  |
|  |  | RPSQQNPQAQ | 1153.55 | 1152.54 | 1152.56 | 78 |  |  |
|  |  | VRVPVPQLQPQNPSQQQPQEQVPL | 2734.40 | 2733.39 | 2733.46 | 61 |  |  |
|  | *Gli-α-12* | CCQHLW | 903.34 | 902.33 | 902.35 | 31 | MKTFLILALLAIVATTATTAVRVPVPQLQPQNPSQQQPQEQVPLVQQQQFLGQQQPFPPQQPYPQPQPFPSQQPYLQLQPFPQPQLPYSQPQPFRPQQPYPQPQPQYSQPQQPISQQQQQHQQQQQQQQQQPQQQQQQQILQQILQQQLIPCMDVVLQQHNIAHGRSQVLQQSTYQLLQELCCQHLWQIPEQSQCQAIHNVVHAIILHQQQKQQQQQPSSQVSFQQPQQQYPLGQGSFRPSQQNPQAQGSVQPQQLPQFEEIRNLALQTLPAICNVYIPPYCTITPFGIFGTN | 31.40% |
|  |  | IAHGRSQVL | 980.54 | 979.54 | 979.56 | 44 |  |  |
|  |  | LWQIPEQSQCQA | 1487.75 | 1486.74 | 1486.69 | 1 |  |  |
|  |  | NIAHGRSQVL | 1094.58 | 1093.58 | 1093.60 | 72 |  |  |
|  |  | QLPQFEEIRNL | 1386.71 | 1385.70 | 1385.73 | 51 |  |  |
|  |  | QPYPQPQP | 954.46 | 953.45 | 953.46 | 21 |  |  |
|  |  | RPQQPYPQPQPQ | 1463.71 | 1462.70 | 1462.73 | 75 |  |  |
|  |  | RPQQPYPQPQPQY | 1626.77 | 1625.76 | 1625.80 | 75 |  |  |
|  |  | RPSQQNPQ | 954.46 | 953.45 | 953.47 | 58 |  |  |
|  |  | RPSQQNPQAQ | 1153.55 | 1152.54 | 1152.56 | 78 |  |  |
|  |  | VRVPVPQLQPQNPSQQQPQEQVPL | 2734.40 | 2733.39 | 2733.46 | 61 |  |  |
| 4 | *Gli-α-8* | IAHGRSQVL | 980.50 | 979.49 | 979.56 | 42 | MKTFLILALLAIVATTATTAVRVPVPQLQPQNPSQQQPQEQVPLVQQQQFLGQQQPFPPQQPYPQPQPFPSQQPYLQLQPFPQPQLPYSQPQPFRPQQPYPQPQPQYSQPQQPISQQQQQQQQQQQQQEQQILQQILQQQLIPCMDVVLQQHNIAHGRSQVLPQSTYQLLQELCCQHLWQIPEQSQCQAIHNVVHAIILHQQQKQQQQPSSQVSFQQSQQQYPLGQGSFRPSQQNPQAQGSVQPQQLPQFEEIRNLALQTLPAMCNVYIPPYCTIAPFGIFGTN | 18.66% |
|  |  | NIAHGRSQVL | 1094.53 | 1093.53 | 1093.60 | 56 |  |  |
|  |  | NPQAQGSVQPQ | 1153.50 | 1152.49 | 1152.55 | 16 |  |  |
|  |  | QLPQFEEIRNL | 1386.64 | 1385.64 | 1385.73 | 65 |  |  |
|  |  | QPQQLPQFEEIRN | 1626.69 | 1625.68 | 1625.82 | 3 |  |  |
|  |  | QQHNIAHGR | 1060.47 | 1059.46 | 1059.53 | 66 |  |  |
|  |  | QQHNIAHGRSQVL | 1487.68 | 1486.67 | 1486.78 | 78 |  |  |
|  |  | RPQQPYPQPQPQ | 1463.64 | 1462.63 | 1462.73 | 100 |  |  |
|  |  | RPQQPYPQPQPQY | 1626.69 | 1625.68 | 1625.80 | 85 |  |  |
|  |  | RPSQQNPQAQ | 1153.50 | 1152.49 | 1152.56 | 80 |  |  |
|  |  | RPSQQNPQAQGSVQPQ | 1749.74 | 1748.73 | 1748.86 | 122 |  |  |
|  |  | RPSQQNPQAQGSVQPQQLPQFEEI | 2734.27 | 2733.26 | 2733.35 | 7 |  |  |
| 5 | *Gli-α-9* | IAHGRSQVL | 980.52 | 979.51 | 979.56 | 46 | MKTFLILALLAIVATTATTAVRVPVPQLQPQNPSQQQPQEQVPLVQQQQFLGQQQPFPPQQPYPQPQPFPSQQPYLQLQPFPQPQLPYSQPQPFRPQQPYPQPQPQYSQPQQPISQQQQQQQQQQQQQQQQEQQILDQILQQQLIPCMDVVLWQHNIAHGRSQVLPQSTYQLLQELCCQHLWQIPEQSQCQAIHNVVHAIILHQQQKQQQQPSSQVSFQQSQQQYPLGQGSFRPSQQNPQAQGSVQPHQLPQFEEIRNLALQTLPAMCNVYIPPYCTIAPFGIFGTN | 22.74% |
|  |  | LWQIPEQSQCQA | 1487.71 | 1486.70 | 1486.69 | 1 |  |  |
|  |  | NIAHGRSQVL | 1094.56 | 1093.55 | 1093.60 | 64 |  |  |
|  |  | QLPQFEEIRNL | 1386.67 | 1385.67 | 1385.73 | 53 |  |  |
|  |  | QPYPQPQP | 954.43 | 953.43 | 953.46 | 23 |  |  |
|  |  | RPQQPYPQPQPQ | 1463.67 | 1462.66 | 1462.73 | 91 |  |  |
|  |  | RPQQPYPQPQPQY | 1626.73 | 1625.72 | 1625.80 | 80 |  |  |
|  |  | RPSQQNPQ | 954.43 | 953.43 | 953.47 | 45 |  |  |
|  |  | RPSQQNPQAQ | 1153.52 | 1152.51 | 1152.56 | 78 |  |  |
|  |  | VRVPVPQLQPQNPSQQQPQEQVPL | 2734.34 | 2733.34 | 2733.46 | 73 |  |  |
| 6 | *Gli-α-10* | CCQHLW | 903.30 | 902.30 | 902.35 | 32 | MKTFLILALLAIVATTATTAVRVPVPQLQPQNPSQQQPQEQVPLVQQQQFLGQQQPFPPQQPYPQPQPFPSQQPYLQLQPFPQPQLPYSQPQPFRPQQPYPQPQPQYSQPQQPISQQQQQQQQQQQQQQQQQQQQQQQQQQPQQQQQQILQQILQQQLIPCMDVVLQQHNIAHGRSQVLQQSTYQLLQELCCQHLWQIPEQSQCQAIHNVVHAIILHQQQKQKQQQPSSQVSFQQPQQQYPLGQGSFRPSQQNPQAQGSVQPQQLPQFEEIRNLALQTLPAMCNVYIPPYCTIAPFGIFGTN | 15.23% |
|  |  | QLPQFEEIRNL | 1386.64 | 1385.64 | 1385.73 | 29 |  |  |
|  |  | RPQQPYPQPQPQ | 1463.64 | 1462.63 | 1462.73 | 12 |  |  |
|  |  | RPQQPYPQPQPQY | 1626.69 | 1625.68 | 1625.80 | 54 |  |  |
|  |  | RPSQQNPQAQGSVQPQQLPQFEEI | 2734.26 | 2733.25 | 2733.35 | 5 |  |  |
|  |  | RPSQQNPQAQGSVQPQQLPQFEEIRNL | 3117.34 | 3116.33 | 3116.58 | 104 |  |  |
|  | *Gli-α-11* | CCQHLW | 903.30 | 902.30 | 902.35 | 32 | MKTFLILALLAIVATTATTAVRVPVPQLQPQNPSQQQPQEQVPLVQQQQFLGQQQPFPPQQPYPQPQPFPSQQPYLQLQPFPQPQLPYSQPQPFRPQQPYPQPQPQYSQPQQPISQQQQQQQQQQQQQQQQQQQQQQQQPQQQQQQILQQILQQQLIPCMDVVLQQHNIAHGRSQVLQQSTYQLLQELCCQHLWQIPEQSQCQAIHNVVHAIILHQQQKQKQQQPSSQVSFQQPQQQYPLGQGSFRPSQQNPQAQGSVQPQQLPQFEEIRNLALQTLPAICNVYIPPYCTITPFGIFGTN | 15.33% |
|  |  | QLPQFEEIRNL | 1386.64 | 1385.64 | 1385.73 | 29 |  |  |
|  |  | RPQQPYPQPQPQ | 1463.64 | 1462.63 | 1462.73 | 12 |  |  |
|  |  | RPQQPYPQPQPQY | 1626.69 | 1625.68 | 1625.80 | 54 |  |  |
|  |  | RPSQQNPQAQGSVQPQQLPQFEEI | 2734.26 | 2733.25 | 2733.35 | 5 |  |  |
|  |  | RPSQQNPQAQGSVQPQQLPQFEEIRNL | 3117.34 | 3116.33 | 3116.58 | 104 |  |  |
| 7 | *Gli-α-13* | CCQHLW | 903.33 | 902.33 | 902.35 | 26 | MKTFLILALLAIVATTATTAVRVPVPQLQPQNPSQQQPQEQVPLVQQQQFLGQQQPFPPQQPYPQPQPFPSQQPYLQLQPFPQPQLPYSQPQPFRPQQPYPQPQPQYSQPQQPISQQQQQQQQQQQQQEQQEQQILQQILQQQLIPCMDVVLQQHNIAHGRSQVLQQSTYQLLQELCCQHLWQIPEQSQCQAIHNVVHAIILHQQQKQQQQPSSQVSFQQPQQQYPLGQGSFRPSQQNPQAQGSVQPQQLPQFEEIRNLALQTLPAMCNVYIPPYCTIAPFGIFGTN | 21.95% |
|  |  | NPQAQGSVQPQ | 1153.54 | 1152.53 | 1152.55 | 5 |  |  |
|  |  | QLPQFEEIRNL | 1386.70 | 1385.69 | 1385.73 | 43 |  |  |
|  |  | QPQQLPQFEEIRN | 1626.75 | 1625.74 | 1625.82 | 3 |  |  |
|  |  | QPYPQPQP | 954.45 | 953.44 | 953.46 | 13 |  |  |
|  |  | QQHNIAHGR | 1060.51 | 1059.50 | 1059.53 | 71 |  |  |
|  |  | RPQQPYPQPQPQ | 1463.69 | 1462.69 | 1462.73 | 101 |  |  |
|  |  | RPQQPYPQPQPQY | 1626.75 | 1625.74 | 1625.80 | 90 |  |  |
|  |  | RPSQQNPQ | 954.45 | 953.44 | 953.47 | 13 |  |  |
|  |  | RPSQQNPQAQ | 1153.54 | 1152.53 | 1152.56 | 65 |  |  |
|  |  | RPSQQNPQAQGSVQPQ | 1749.81 | 1748.80 | 1748.86 | 101 |  |  |
| 8 | *Gli-γ-1* | AQIPRQL | 825.47 | 824.46 | 824.49 | 27 | MKTLFILTILAMATTIATANMQVDPSGQVQWPQQQPFRQPQQPFYQQPQQTFPQPQQTFPHQPQQQFPQPQQPQQQFPQPQQPQQPFPQPQQAQLPFPQQPQQPFPQPQQPQQPFPQSQQPQQPFPQPQQPQQSFPQQQQPLIQPYLQQQMNPCKNYLLQQCNPVSLVSSLVSMILPRSDCQVMQQQCCQQLAQIPRQLQCAAIHSVVHSIIMQQEQQQGIQILRPLFQLVQGQGIIQPQQPAQYEVIRSLVLRTLPNMCNVYVRPDCSTINAPFASIVAGISGQ | 17.54% |
|  |  | EVIRSLVL | 928.55 | 927.54 | 927.58 | 27 |  |  |
|  |  | ILPRSDCQVM | 1218.55 | 1217.54 | 1217.59 | 40 |  |  |
|  |  | RTLPNMCNVY | 1267.55 | 1266.54 | 1266.58 | 60 |  |  |
|  |  | VLRTLPNMCNVY | 1479.69 | 1478.68 | 1478.74 | 55 |  |  |
|  |  | VRPDCSTINAPF | 1376.61 | 1375.60 | 1375.66 | 61 |  |  |
|  |  | VSMILPRSDCQVM + Oxidation (M) | 1551.67 | 1550.67 | 1550.73 | 7 |  |  |
| 9 | *Gli-γ-1* | AQIPRQL | 825.49 | 824.49 | 824.49 | 28 | MKTLFILTILAMATTIATANMQVDPSGQVQWPQQQPFRQPQQPFYQQPQQTFPQPQQTFPHQPQQQFPQPQQPQQQFPQPQQPQQPFPQPQQAQLPFPQQPQQPFPQPQQPQQPFPQSQQPQQPFPQPQQPQQSFPQQQQPLIQPYLQQQMNPCKNYLLQQCNPVSLVSSLVSMILPRSDCQVMQQQCCQQLAQIPRQLQCAAIHSVVHSIIMQQEQQQGIQILRPLFQLVQGQGIIQPQQPAQYEVIRSLVLRTLPNMCNVYVRPDCSTINAPFASIVAGISGQ | 12.98% |
|  |  | EVIRSLVL | 928.57 | 927.57 | 927.58 | 32 |  |  |
|  |  | ILPRSDCQVM | 1218.59 | 1217.58 | 1217.59 | 41 |  |  |
|  |  | QLAQIPRQ | 953.55 | 952.54 | 952.55 | 7 |  |  |
|  |  | RTLPNMCNVY | 1267.58 | 1266.58 | 1266.58 | 69 |  |  |
|  |  | VLRTLPNMCNVY | 1479.73 | 1478.73 | 1478.74 | 32 |  |  |
| 10 | *Gli-γ-1* | AQIPRQL | 825.44 | 824.44 | 824.49 | 28 | MKTLFILTILAMATTIATANMQVDPSGQVQWPQQQPFRQPQQPFYQQPQQTFPQPQQTFPHQPQQQFPQPQQPQQQFPQPQQPQQPFPQPQQAQLPFPQQPQQPFPQPQQPQQPFPQSQQPQQPFPQPQQPQQSFPQQQQPLIQPYLQQQMNPCKNYLLQQCNPVSLVSSLVSMILPRSDCQVMQQQCCQQLAQIPRQLQCAAIHSVVHSIIMQQEQQQGIQILRPLFQLVQGQGIIQPQQPAQYEVIRSLVLRTLPNMCNVYVRPDCSTINAPFASIVAGISGQ | 21.75% |
|  |  | EVIRSLVL | 928.52 | 927.51 | 927.58 | 33 |  |  |
|  |  | ILPRSDCQVM | 1218.52 | 1217.51 | 1217.59 | 38 |  |  |
|  |  | LQQCNPVSLVSS | 1331.59 | 1330.58 | 1330.66 | 1 |  |  |
|  |  | RTLPNMCNVY | 1267.51 | 1266.50 | 1266.58 | 61 |  |  |
|  |  | VLRTLPNMCNVY | 1479.64 | 1478.64 | 1478.74 | 54 |  |  |
|  |  | VRPDCSTINAPF | 1376.57 | 1375.57 | 1375.66 | 58 |  |  |
|  |  | VSMILPRSDCQVM | 1535.63 | 1534.62 | 1534.73 | 38 |  |  |
| 11 | *Gli-γ-2* | RTLPNMCNVY | 1267.54 | 1266.53 | 1266.58 | 68 | MKTLFILTILAMATTIATANMQVDPSGQVQWPQQQPFRQPQQPFYQQPQQTFPHQPQQQFPQPQQSQQQFPQPQQPQQPFPQPQQAQLPFPQQPQQPFPQPQQPQQPFPQSQQPQQPFPQPQQPQQSFPQQQQPLIQPYLQQQMNPCKNYLLQQCNPVSLVSSLVSMILPRSDCQVIQQQCCQQLAQIPRQLQCAAIHSVVHSIIMQQEQQQGIQILRPLFQLVQGQGIIQPQQPAQYEVIRSLVLRTLPNMCNVYVRPDCSTINAPFASIVAGISGQ | 7.94% |
|  |  | VRPDCSTINAPF | 1376.60 | 1375.59 | 1375.66 | 62 |  |  |
| 12 | *Gli-γ-3* | GIQIMRPL | 927.52 | 926.51 | 926.54 | 13 | MKTLLILTIIAVALTTTTANIQVDPSGQVQWPQQQQPFPQPQQPFSQQPQQIFPQPQQTFPHQPQQAFPQPQQTFPHQPQQQFPQPQQPQQPFPQQPQQQFPQPQQPQQPFLQQPQQQFPQPQKPQQPFPQPQQPQLPFPQQPQQPFPQPQQPQQPFPQLQQPQQPLPQPQQPQQPFPQQQQPLIQPYLQQQMNPCKNYLLQQCNPVSLVSSLVSMILPRSDCQVMRQQCCQQLAQIPQQLQCAAIHGVVHSIIMQQEQQEQQQQQQQQQQQQQQGIQIMRPLFQLVQGQGIIQPQQPAQLEVIRSLVLGTLPTMCNVFVPPECSTTKAPFASIVADIGGQ | 10.85% |
|  |  | ILPRSDCQVM | 1218.56 | 1217.55 | 1217.59 | 18 |  |  |
|  |  | LQQQMNPCKNY | 1423.59 | 1422.58 | 1422.64 | 45 |  |  |
|  |  | RQQCCQQL | 1120.46 | 1119.45 | 1119.49 | 49 |  |  |
| 13 | *TRIUR3_09156* (*Avenin-3*) | AHISEPSRCPAIH | 1474.66 | 1473.65 | 1473.71 | 30 | MKIFLVFALLVVSTIITTATAQLDPSIHVQERPQQSFLQQQPLTQQQPFPLQEPQQPLFQQQQPYPQQSLPQQQLPQQHLFPQQPPQQQFPQQMPFPHQQQIFPQQQQPPQQQPFYQYQQPLTQQPYPQEQALPQQQPSVEENQQLNLCKEFLLQQCNPEEKLSLLQSVIPFLRPKTSQQNNCQLKRQQCCRQLAHISEPSRCPAIHNTVHAIIMQQQQQQQQQQHVDRGFVQPQPQQLGQGMPMQPQHQLGQGLSLPQQLAQFKLVRLLVIQTLPMLCNVHVPSDCYTISAPFGGITAYNGGQ | 20.40% |
|  |  | AHISEPSRCPAIHNTVH | 1925.86 | 1924.85 | 1924.93 | 89 |  |  |
|  |  | LRPKTSQQNNCQL | 1586.74 | 1585.73 | 1585.80 | 2 |  |  |
|  |  | PPQQQPF | 841.40 | 840.39 | 840.41 | 38 |  |  |
|  |  | QQQQQQQQQQHVDRGF | 2010.86 | 2009.85 | 2009.94 | 60 |  |  |
|  |  | VQERPQQSF | 1118.51 | 1117.51 | 1117.55 | 45 |  |  |
| 14 | *TuA3-538a* (LMW-GS, KM085281) | ARSQMLQQSICH | 1458.58 | 1457.57 | 1457.69 | 48 | ISQQQQAPPFSQQQQPPFSQQQQPPFSQQQQSPFSQQQQQPPFAQQQQPPFSQQPPISQQQQPPFSQQQQPQFSQQQQAPYSQQQQPPYSQQQQPPFSQQQQPPFSQQQQQPPFTQQQQPPFSQQPPISQQQQPPFSQQQQPPFSQQQQIPVIHPSVLQQLNPCKVFLQQQCIPVAMQRCLARSQMLQQSICHVMQQQCCQQLRQIPEQSRHESIRAIVYSIILQQQQQQQQQQQGQSIIQYQQQQPQQLGQCVSQPQQELQQQLGQQPQQQQLAHGTFLQPHQIAQLEVMTSIALRNLPTMCSVNVPLYETTTSVPLGVGIGVGVY | 13.76% |
|  |  | ESIRAIVY | 950.46 | 949.45 | 949.52 | 30 |  |  |
|  |  | QQLNPCKVF | 1133.49 | 1132.48 | 1132.57 | 54 |  |  |
|  |  | RNLPTMC | 891.35 | 890.35 | 890.41 | 38 |  |  |
|  |  | RQIPEQSRH | 1150.52 | 1149.51 | 1149.60 | 46 |  |  |
|  |  | RQIPEQSRHESIR | 1635.74 | 1634.73 | 1634.86 | 54 |  |  |
|  |  | RQIPEQSRHESIRAIVY | 2081.95 | 2080.95 | 2081.11 | 15 |  |  |
