# Supplementary material for: Genome-, Transcriptome- and Proteome-Wide Analyses of the Gliadin Gene Families in Triticum urartu
Source: PLoS One. 2015 Jul 1;10(7):e0131559. doi: 10.1371/journal.pone.0131559 (PMC4489009; doi:10.1371/journal.pone.0131559)
Supplement: S8 Table — (DOCX) [file pone.0131559.s009.docx]

**S8 Table. LC-MS/MS identification of the protein spots from the LMW-GS fractions of *T. urartu* accession PI428198 after separation via 2-DE.**

| **Spot** | **Matching gene** | **Mass spectrum** | **MH^+^** | **Charge** | **XC** | **Predicted gliadin/LMW-GS protein sequence** | **Coverage** |
| --- | --- | --- | --- | --- | --- | --- | --- |
| 15 | *TuA3-576c* (LMW-GS, KM085304) | -.ARSQML.- | 705.37 | 1 | 1.51 | ISQQQQPPPFSEQQQPPFSQKQQPPFSQQQQSPFSQQQQQPPFSQQQQPPFSQQPPISQQQQPPFSQQQQPQFSQQQQPPYSQQQQPPYSQQQQPPFSQQQQPPFSQQQQQPPISQQQQQQQQQQPFTQQQQPPFSQQPPISQQQQPPFSQQQRPPFSQQQQIPVIHPSVLQQLNPCKVFLQQQCIPVAMQRCLARSQMLQQSICHVMQQQCCQQLRQIPEKSRHESIRAIIYSIILQQQQQQQQQQQGQSIIQYQQQQPQQLGQCVSQPLQQLQQQLGQQPQQQQLAYQIAQLEVMTSIALRTLPTMCSVNVPLYETTTSVPLGVGIGVGVY | 28.23% |
|  |  | -.AYQIAQL.- | 806.44 | 1 | 1.78 |  |  |
|  |  | -.QQLNPCKVF.- | 1133.58 | 2 | 2.33 |  |  |
|  |  | -.SQKQQPPF.- | 959.50 | 1 | 1.52 |  |  |
|  |  | -.SQQQQPPF.- | 959.46 | 1 | 1.77 |  |  |
|  |  | -.SQQQQPPY.- | 975.45 | 1 | 1.69 |  |  |
|  |  | -.SQQQQSPF.- | 949.44 | 1 | 1.67 |  |  |
|  |  |  |  |  |  |  |  |
| 16 | *TuA3-520* (LMW-GS, KM085275) | F.LQPHQIAQF.E | 1081.58 | 2 | 2.57 | ISQQQQQQPFPQQQQPPFSQQQEPPYSQQQQPPFSQQQQSPFSQQQQQPPFLQQHQPPFSQQPPISQQQQPPFSQQQQPPFSQQQQPPFSQQQQQPPFSQQQQQQQPPFSQQQQPPFSQQPPISQKQQPPFSQQQQPPFSQQQQIPVIHPSVLQQLNPCKVFLQQQCIPVAMQRCLARSQMLQQRICHMMQQQCCQQLRQIPGQSRHESIRAIIYSIILQQQQQQQQQQQQQQVQSIIQAQQQQPQQLGQCVSQPQQQSQQQLGQQPQQQQLAQGTFLQPHQIAQFEVMTSIALRTLPMMCNVNVPLYGTTTSAPFGVGTGVGAY | 31.08% |
|  |  | F.LQPHQIAQFEVM.T | 1440.73 | 2 | 2.67 |  |  |
|  |  | F.SQQQQIPVIHPSVL.Q | 1573.87 | 2 | 2.17 |  |  |
|  |  | H.ESIRAIIY.S | 964.55 | 1 | 1.50 |  |  |
|  |  | I.PVIHPSVL.Q | 861.52 | 1 | 1.71 |  |  |
|  |  | L.GQCVSQPQQQSQQQLGQQPQQQQL.A | 2779.32 | 3 | 3.23 |  |  |
|  |  | L.GQQPQQQQL.A | 1054.53 | 2 | 2.27 |  |  |
|  |  | L.QQLNPCKVF.L | 1133.58 | 2 | 2.50 |  |  |
|  |  | Q.PPFSQQQQIPVIHPSVL.Q | 1915.04 | 2 | 2.69 |  |  |
|  |  | Q.SIIQAQQQQPQQL.G | 1509.80 | 2 | 2.22 |  |  |
|  |  | Q.SQQQLGQQPQQQQL.A | 1638.82 | 2 | 2.00 |  |  |
