# Supplementary material for: Genome-, Transcriptome- and Proteome-Wide Analyses of the Gliadin Gene Families in Triticum urartu
Source: PLoS One. 2015 Jul 1;10(7):e0131559. doi: 10.1371/journal.pone.0131559 (PMC4489009; doi:10.1371/journal.pone.0131559)
Supplement: S9 Table — (DOCX) [file pone.0131559.s010.docx]

**S9 Table. The level of nucleotide sequence identity between the gliadin genes of *T. urartu* accession PI428198 and previously reported genes/allelic variants.**

| **Gene** | **Accession No.** | **Type** | **Common wheat** | |  |  | **Wheat relatives** | | |
| --- | --- | --- | --- | --- | --- | --- | --- | --- | --- |
|  |  |  | **Accession No.** | **Identity** |  |  | **Accession No.** | **Taxonomy** | **Identity** |
| ***Gli-α-1*** | KP280186 | alpha | EU018276 | 98% |  |  | DQ296197 | *Triticum turgidum subsp. durum* | 98% |
|  |  |  |  |  |  |  | JX828226 | *Aegilops tauschii* | 97% |
|  |  |  |  |  |  |  | JX828193 | *Triticum monococcum* | 96% |
| ***Gli-α-2*** | KP280187 | alpha | EU018276 | 98% |  |  | JX828226 | *Aegilops tauschii* | 98% |
|  |  |  |  |  |  |  | FJ441091 | *Triticum monococcum* | 97% |
| ***Gli-α-3*** | KP280197 | alpha | EU018276 | 96% |  |  | FJ441091 | *Triticum monococcum* | 97% |
|  |  |  |  |  |  |  | HM188550 | *Aegilops tauschii* | 97% |
| ***Gli-α-4*** | KP280198 | alpha | KC715913 | 96% |  |  | JX828228 | *Aegilops tauschii* | 97% |
|  |  |  |  |  |  |  | DQ401700 | *Triticum monococcum* | 97% |
|  |  |  |  |  |  |  | JQ340386 | *Triticum dicoccoides* | 97% |
| ***Gli-α-5*** | KP280199 | alpha | EF561275 | 99% |  |  | KC421083 | *Triticum monococcum* | 99% |
|  |  |  |  |  |  |  | DQ140352 | *Triticum turgidum subsp. Dicoccoides* | 99% |
|  |  |  |  |  |  |  | JX828221 | *Aegilops tauschii* | 97% |
| ***Gli-α-6*** | KP280200 | alpha | KC715890 | 98% |  |  | JX828221 | *Aegilops tauschii* | 98% |
|  |  |  |  |  |  |  | KC421078 | *Triticum monococcum* | 97% |
|  |  |  |  |  |  |  | JX275673 | *Triticum dicoccoides* | 98% |
| ***Gli-α-7*** | KP280201 | alpha | EU680852 | 98% |  |  | JQ340386 | *Triticum dicoccoides* | 98% |
|  |  |  |  |  |  |  | JX828228 | *Aegilops tauschii* | 98% |
|  |  |  |  |  |  |  | JN831382 | *Triticum monococcum* | 98% |
| ***Gli-α-8*** | KP280202 | alpha | KJ137236 | 98% |  |  | KJ410473 | *Aegilops speltoides* | 99% |
|  |  |  |  |  |  |  | FJ441086 | *Triticum monococcum* | 99% |
|  |  |  |  |  |  |  | DQ140352 | *Triticum turgidum subsp. Dicoccoides* | 98% |
| ***Gli-α-9*** | KP280203 | alpha | EF561283 | 98% |  |  | DQ140352 | *Triticum turgidum subsp. Dicoccoides* | 98% |
|  |  |  |  |  |  |  | KC421083 | *Triticum monococcum* | 97% |
| ***Gli-α-10*** | KP280176 | alpha | KC715913 | 96% |  |  | JX828228 | *Aegilops tauschii* | 97% |
|  |  |  |  |  |  |  | DQ401700 | *Triticum monococcum* | 97% |
|  |  |  |  |  |  |  | JQ340382 | *Triticum dicoccoides* | 97% |
| ***Gli-α-11*** | KP280177 | alpha | KC715913 | 97% |  |  | JX828228 | *Aegilops tauschii* | 98% |
|  |  |  |  |  |  |  | DQ401700 | *Triticum monococcum* | 97% |
|  |  |  |  |  |  |  | JQ340386 | *Triticum dicoccoides* | 97% |
| ***Gli-α-12*** | KP280178 | alpha | EU680852 | 98% |  |  | JX828219 | *Aegilops tauschii* | 98% |
|  |  |  |  |  |  |  | JQ340388 | *Triticum dicoccoides* | 98% |
|  |  |  |  |  |  |  | FJ441088 | *Triticum monococcum* | 98% |
| ***Gli-α-13*** | KP280179 | alpha | EF561283 | 99% |  |  | DQ140352 | *Triticum turgidum subsp. Dicoccoides* | 99% |
|  |  |  |  |  |  |  | JX828195 | *Triticum monococcum* | 98% |
| ***Gli-α-14*** | KP280180 | alpha | JX141494 | 96% |  |  | FJ441091 | *Triticum monococcum* | 97% |
|  |  |  |  |  |  |  | HM188550 | *Aegilops tauschii* | 97% |
| ***Gli-α-15*** | KP280181 | alpha | JN831404 | 97% |  |  | JX275677 | *Triticum dicoccoides* | 98% |
|  |  |  |  |  |  |  | GQ999810 | *Triticum turgidum subsp.durum* | 98% |
| ***Gli-α-16*** | KP280182 | alpha | KC715909 | 98% |  |  | JX275677 | *Triticum dicoccoides* | 98% |
| ***Gli-α-17*** | KP280183 | alpha | X54517 | 97% |  |  | JX275686 | *Triticum dicoccoides* | 97% |
|  |  |  |  |  |  |  | GQ999810 | *Triticum turgidum subsp.durum* | 96% |
|  |  |  |  |  |  |  | JX828218 | *Aegilops tauschii* | 96% |
| ***Gli-α-18*** | KP280184 | alpha | X54517 | 97% |  |  | JX275686 | *Triticum dicoccoides* | 97% |
|  |  |  |  |  |  |  | GQ999810 | *Triticum turgidum subsp.durum* | 97% |
|  |  |  |  |  |  |  | JX828218 | *Aegilops tauschii* | 96% |
| ***Gli-α-19*** | KP280185 | alpha | X54517 | 97% |  |  | JX275669 | *Triticum dicoccoides* | 96% |
|  |  |  |  |  |  |  | HM188550 | *Aegilops tauschii* | 96% |
|  |  |  |  |  |  |  | GQ999810 | *Triticum turgidum subsp.durum* | 96% |
| ***Gli-α-20*** | KP280188 | alpha | JX828233 | 96% |  |  | JX828195 | *Triticum monococcum* | 95% |
|  |  |  |  |  |  |  | HM188550 | *Aegilops tauschii* | 96% |
| ***Gli-α-21*** | KP280189 | alpha | EF561277 | 94% |  |  | GQ999808 | *Triticum turgidum subsp.durum* | 97% |
| ***Gli-α-22*** | KP280190 | alpha |  |  |  |  | FJ441077 | *Triticum monococcum* | 97% |
| ***Gli-α-23*** | KP280191 | alpha | KC715913 | 94% |  |  | JX828228 | *Aegilops tauschii* | 95% |
|  |  |  |  |  |  |  | DQ401700 | *Triticum monococcum* | 95% |
|  |  |  |  |  |  |  | JQ340386 | *Triticum dicoccoides* | 95% |
| ***Gli-γ-1*** | KP280192 | gamma | JX679678 | 99% |  |  | FJ006627 | *Triticum monococcum* | 99% |
| ***Gli-γ-2*** | KP280193 | gamma | KF412608 | 97% |  |  | FJ006627 | *Triticum monococcum* | 97% |
| ***Gli-γ-3*** | KP280194 | gamma | JX679673 | 99% |  |  | JQ269808 | *Triticum monococcum* | 94% |
| ***Gli-ω-1*** | KP280195 | omega | KC716067 | 87% |  |  | JX295577 | *Aegilops tauschii* | 86% |
| ***Gli-ω-2*** | KP280196 | omega | AB059812 | 97% |  |  | JX295577 | *Aegilops tauschii* | 77% |
